# A multiscale *in situ* time-resolved study of the nano- to millisecond structural dynamics during protein crystallization

**DOI:** 10.1107/S160057672500353X

**Published:** 2025-05-29

**Authors:** Christian Beck, Ilaria Mosca, Laura M. Miñarro, Benedikt Sohmen, Cara Buchholz, Ralph Maier, Lara Franziska Reichart, Anna Carlotta Grundel, Famke Bäuerle, Roody Nasro, Hadra Banks, Simon Christmann, Kai-Florian Pastryk, Bela Farago, Orsolya Czakkel, Sylvain Prévost, Alexander Gerlach, Marco Grimaldo, Felix Roosen-Runge, Olga Matsarskaia, Frank Schreiber, Tilo Seydel

**Affiliations:** a Institute of Applied Physics University of Tübingen 72076Tübingen Germany; b Institut Max von Laue–Paul Langevin, 38042Grenoble Cedex 9, France; c Université Grenoble Alpes, 38400Saint-Martin-d’Hères, France; d Fakultät für Physik und Astronomie Universität Heidelberg Im Neuenheimer Feld 226 69120Heidelberg Germany; e Division of Physical Chemistry Lund University Naturvetarvägen 22 22362Lund Sweden; Uppsala University, Sweden; The European Extreme Light Infrastucture, Czechia

**Keywords:** *in situ* protein crystallization, diffusion, structural dynamics, small-angle neutron scattering, neutron spectroscopy

## Abstract

Protein crystallization is key to determining the structure of proteins at atomic resolution. It can occur naturally, including in pathological pathways, for instance with aquaporin and γ-crystallin proteins. A fundamental understanding of the underlying crystallization process is both technologically and biologically relevant. A multitechnique approach is employed here to investigate protein crystallization *in situ*, allowing us to assess the evolution of the liquid suspension and crystallite structure as well as protein diffusion during the crystallization process. The wide range of methods probe the sample on ångström to millimetre length scales, accessing nanosecond to millisecond dynamics information while acquiring data with minute-timescale kinetic resolution during crystallization. This process takes several hours from an initial state of monomers or small clusters until the presence of large crystallites. Employing neutron spectroscopy allows us to distinguish different crystallization pathways and to reveal the presence of coexisting clusters during the entire crystallization process. We demonstrate the multitechnique approach on human serum albumin (HSA) proteins crystallized from aqueous solution in the presence of LaCl_3_. For this system, the crystallization kinetics can be consistently described by a sigmoid function across all methods, and the kinetics can be controlled by the salt concentration. Moreover, we compare the HSA–LaCl_3_ model system with the crystallization behavior of β-lactoglobulin–CdCl_2_, which includes a metastable intermediate state.

## Abstract 2

Protein crystallization is a crucial method for structure determination and biological studies, but its pathways remain poorly understood, often relying on trial and error to initiate crystallization. Using a multitechnique approach, this study investigates structural and diffusion properties across various time and length scales to compare different crystallization conditions and systems.

## 1. Introduction

Knowledge of the structural and dynamical properties of biological macromolecules such as proteins, vesicles or nucleic acids constitutes a key element for the comprehension of interactions in biological processes on a molecular level. As a part of this endeavor, the understanding of protein crystallization is crucial for a systematic, reliable and predictable preparation of protein crystals (Durbin & Feher, 1996 ▸; Giegé, 2013 ▸), as well as for understanding their natural occurrence (Schönherr *et al.*, 2018 ▸) and associated pathological malfunctions (Li *et al.*, 2023 ▸; Chhana *et al.*, 2019 ▸; Vekilov *et al.*, 2002 ▸; Pande *et al.*, 2001 ▸). Besides their use to determine the structure of the macromolecules that serve as their building blocks (Kapetanaki *et al.*, 2024 ▸; Jackson *et al.*, 2023 ▸), protein crystals can be used for nanoscale applications in medicine (Hartje & Snow, 2018 ▸) and host foreign molecules allowing catalytic reactions (Kojima *et al.*, 2022 ▸).

Protein crystallization can be a remarkably slow process (Vekilov & Alexander, 2000 ▸; Nanev, 2007 ▸), and successful crystallization in the laboratory mainly results from trial and error (Nanev, 2007 ▸). This trial-and-error process involving numerous days of laboratory work also forms the basis for the crystallization conditions reported in the present study. A better understanding of protein crystallization, with the aim of a future rational determination of crystallization parameters requires, *inter alia*, knowledge of the nanosecond diffusive dynamics during the crystallization process. This knowledge permits the disentanglement of possible dynamic dissociation and phase equilibria between crystallized and dissolved fractions and enables measuring the diffusion in the dissolved fraction. Moreover, it is important to determine whether the crystallization is preceded by small, possibly transient, protein aggregates because by simulations and light scattering experiments transient clusters have been found to play a role in crystal nucleation (Piazza & Iacopini, 2002 ▸; Liu *et al.*, 2009 ▸).

Protein crystallization depends on control parameters such as temperature (Liu *et al.*, 2011 ▸), electric fields (Ray *et al.*, 2024 ▸) or pressure (Suzuki *et al.*, 2002 ▸; Collins *et al.*, 2011 ▸). By changing the protein–protein interactions, crystallization processes can be triggered, for example, by carefully adjusting the concentration of co-solutes in the solution, using phenomena such as the excluded volume effect (Rüffer *et al.*, 2024 ▸) or electrostatic interactions. The presence of salt ions in solution can screen repellent forces (Liang *et al.*, 2024 ▸) or act as salt bridges (Kurihara *et al.*, 2023 ▸; Surfaro *et al.*, 2024 ▸). Previous studies have also shown different crystal structures as a function of the salt concentration (Tsuyuguchi *et al.*, 2018 ▸). The kinetic crystallization process is equally relevant, and distinct dynamical pathways of protein crystallization have been discussed previously, notably based on simulations (Whitelam, 2010 ▸). Real-time investigations with noninvasive techniques allow different pathways to be disentangled (Walla *et al.*, 2023 ▸; Alexandrov & Makoveeva, 2023 ▸; Makoveeva *et al.*, 2024 ▸), such as those associated with one-step nucleation or two-step nucleation passing through a metastable intermediate phase (Sauter *et al.*, 2015*a* ▸). Some approaches focus on individual crystals, *e.g.* optical microscopy (Van Driessche *et al.*, 2007 ▸; Mentges *et al.*, 2024 ▸), atomic force microscopy (Zhai *et al.*, 2024 ▸) and cryo-electron microscopy (Harder *et al.*, 2023 ▸), allowing a detailed picture of the specific pathway to be obtained. In contrast, scattering techniques provide an ensemble average of a large number of particles in the observation volume, having the advantage of being immediately statistically meaningful.

Other important parameters controlling crystallization include the concentration of salt and its valency. Salt-induced crystallization has been intensively studied in several protein systems (Timofeev & Samygina, 2023 ▸; McPherson & Gavira, 2013 ▸; Kundrot, 2004 ▸; Liu *et al.*, 2023 ▸; Zhang, 2017 ▸). While some proteins crystallize in the presence of monovalent salts (Marchenkova *et al.*, 2022 ▸; Fahim *et al.*, 2024 ▸) and can be described with theoretical models (Schmit & Dill, 2010 ▸; Schmit & Dill, 2012 ▸), other protein solutions require multivalent ions to induce protein–protein interactions (Buchholz *et al.*, 2023 ▸) suitable for crystal formation. We emphasize that the presence of multivalent salts in the protein solution does not necessarily imply protein crystallization but can also result in a rich phase diagram (Matsarskaia *et al.*, 2020 ▸), including re-entrant phase separation (Braun *et al.*, 2018 ▸; Matsarskaia *et al.*, 2018 ▸), protein aggregation (Beck *et al.*, 2021 ▸; Grimaldo *et al.*, 2015*b* ▸; Soraruf *et al.*, 2014 ▸) or liquid–liquid phase separation (Braun *et al.*, 2017 ▸; Mosca *et al.*, 2024 ▸; Surfaro *et al.*, 2023 ▸). In the present work, we address crystallization driven by di- and trivalent salts.

X-ray and neutron scattering techniques enable the investigation of samples at nanometre to atomic resolution and, with sufficient beam brightness, the time-dependent structural evolution of the crystals can be followed *in situ* (Longo *et al.*, 2021 ▸). Unlike techniques that use visible light, such as optical microscopy (Sazaki *et al.*, 2012 ▸; Van Driessche *et al.*, 2007 ▸) or dynamic light scattering (DLS) (Proteau *et al.*, 2010 ▸), X-rays and neutrons can be used to investigate turbid and opaque samples. Moreover, X-rays and neutrons access spatial correlations associated with molecular length scales.

The nuclear-isotope-dependent neutron scattering cross-sections, which are particularly large and different for ^1^H and ^2^H = D, offer a unique complementary view allowing one to reveal the positions of hydrogen atoms in crystal structures (Drago *et al.*, 2024 ▸), as well as to obtain unique dynamic information via the self and distinct parts in Van Hove correlation functions (Van Hove, 1954 ▸).

Nuclear spin incoherent neutron spectroscopy measures the ensemble-averaged single-particle self-correlation function, thus giving access to the self-diffusion (Van Hove, 1954 ▸). This self-diffusion provides information on the hydrodynamic size of a protein aggregate via the Stokes–Einstein relation, which for translational diffusion of spherical particles, *D*_s,t_, at infinite dilution reads 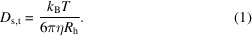
Here *k*_B_ is the Boltzmann constant, *T* is the temperature, η is the solvent viscosity and *R*_h_ is the hydrodynamic radius. This relation can be generalized to account for crowding and rotational diffusion (Roosen-Runge *et al.*, 2011 ▸).

High-resolution neutron spectroscopy permits the identification of even short-lived clusters through their self-diffusion due to its observation or coherence time, which results from its energy resolution and, depending on the spectrometer employed, can range from picoseconds to hundreds of nanoseconds (Wang *et al.*, 2024 ▸; Grimaldo *et al.*, 2019 ▸; Osti *et al.*, 2024 ▸). In our work, we measure this self-diffusion by neutron backscattering spectroscopy (NBS). Conversely, coherent neutron spectroscopy measures the ensemble-averaged pair correlation function, giving access to the collective diffusion function and thus to structural dynamics, depending on both the hydrodynamic function and static structure factor of a protein solution (Beenakker & Mazur, 1984 ▸; Banchio & Nägele, 2008 ▸). In our work, we measure this collective diffusion, corresponding to short-time diffusion in colloid physics terms (Nägele, 1996 ▸), by neutron spin echo (NSE) spectroscopy. Section 3 details on how these quantities are derived. In contrast to X-ray scattering, cold and thermal neutron scattering bears the additional advantage of not causing any radiation damage to fragile biological samples. Employing cold neutrons with energies on the order of 2 meV in our experiments, *i.e.* energies of approximately three orders of magnitude lower than the energy required to break covalent chemical bonds, allowed us to keep the samples for days in the neutron beam without noticeable issues caused by radiation damage.

In the present work, we combine small-angle neutron scattering (SANS) with *in situ* measurements of optical microscopy, DLS and NSE spectroscopy, as well as NBS, to explore the dynamics and kinetics of protein crystallization for two systems. By means of the listed methods, we access the structure, the conformation in solution and crystallite structure, the long-time collective diffusion at small wavevector magnitude *q* (*i.e.* momentum transfer ℏ*q*), the short-time collective diffusion on momentum transfers near the crystallite Bragg peaks, and the short-time self-diffusion. In the long-time limit, with millisecond observation timescale, numerous direct protein–protein interactions, *i.e.* ‘collisions’ in solution, occur in a many-particle system. In contrast, in the short-time limit with nanosecond observation time, these collisions can be neglected, and hydrodynamic as well as electrostatic interactions dominate. The hydrodynamic interactions already slow down the short-time diffusion substantially in a crowded protein solution compared with dilute systems (Roosen-Runge *et al.*, 2011 ▸). Importantly, combining these different types of real-time structural and spectroscopic data, we obtain a rich picture of the diffusive dynamics during the crystallization kinetics on different observation scales, which may help to better understand crystallite growth and crystallization phase behavior.

For a manageable sample parameter space, given the limited access to neutron beams, we restrict ourselves to a fixed protein concentration of human serum albumin (HSA) at *c*_p_ = 75 mg ml^−1^ and choose two salt concentrations (*c*_s_ = 4.5 and 4.785 m*M* LaCl_3_) that are intriguingly close but result in different structures, as will be evidenced by the Bragg peak positions found in diffraction patterns reported in this work. Moreover, we study bovine β-lactoglobulin (BLG) at *c*_p_ = 84.4 mg ml^−1^ with *c*_s_ = 30 m*M* CdCl_2_. With these systems we establish the method framework and explain its use, paving the way for future systematic studies of other systems and elucidating the complex protein crystallization process across substantial time and length scales.

## 2. Materials and methods

### 2.1. Materials

HSA (A9511, batch number: SLCN0120), BLG (L3908, batch: SLCM7980), LaCl_3_ (449830), CdCl_2_ (202908) and D_2_O were purchased from Sigma–Aldrich (Merck KGaA, Darmstadt, Germany). The proteins and salts were used as purchased without further purification.

### 2.2. Sample preparation

Salt stock solutions were prepared in D_2_O with salt concentrations of *c*_LaCl_3__ = 100 m*M* and *c*_CdCl_2__ = 400 m*M*. Protein stock solutions were prepared by dissolving a protein mass *m* in a volume *V* of D_2_O to obtain a solution with the nominal protein concentration 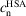
 = *mV*^−1^ = 200 mg ml^−1^ for HSA and 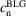
 = *mV*^−1^ = 300 mg ml^−1^ for BLG. The proteins were dissolved in D_2_O without dialysis to circumvent inaccuracies in the final protein concentration, but H/D exchange had been confirmed to be negligible in earlier work (Grimaldo *et al.*, 2015*a* ▸). Residual salts (concentration *c*_res_) in the as-received protein samples may influence the total salt concentration (*c*_s_ = *c*_LaCl_3_/CdCl_2__ + *c*_res_) in the samples, but since all experiments were carried out on the same protein batch, this possible salt concentration offset has no further impact. The protein concentration of the stock solution was subsequently determined by UV–Vis measurements applying an absorption coefficient ɛ_HSA_ = 0.531 l g^−1^ cm^−1^ for HSA (Mendez *et al.*, 2005 ▸) and ɛ_BLG_ = 0.96 l g^−1^ cm^−1^ for BLG (Sober, 1970 ▸). The protein stock solution, pure D_2_O and salt stock solution were mixed in appropriate volumes to match the desired concentrations. No adjustments of pD were performed. All samples were prepared in temperature-stabilized laboratories (*T* = 20°C).

### 2.3. Optical microscopy

Light microscopy studies were performed using the Olympus BX61 microscope with a motorized sample stage located at the Partnership for Soft Condensed Matter (PSCM) laboratory in Grenoble (France). To achieve a larger sample volume, microscope slides with cavities were used. Images were acquired every 30 min in bright-field transmission mode with a 10× magnification, enlarging the observed area by moving the sample stage and changing between two samples positioned on the sample stage. Each tenth acquisition has been analyzed. The time sequences obtained were split with *ImageJ* (Schneider *et al.*, 2012 ▸) using the *Bio-Formats* package (Linkert *et al.*, 2010 ▸) and further analyzed with purpose-written MATLAB (The MathWorks Inc., 2022 ▸) code. Since the microscopy samples were contained between the bottom plate and a slip cover, as opposed to the fully sealed containers used in all other experiments, an H/D exchange of the solvent during the acquisition cannot be ruled out.

### 2.4. Dynamic light scattering

Dynamic light scattering was carried out by repetitive measurements iterating over the angular range between 30° and 150° in 10° steps on an ALV CGS-3 Compact Goniometer System (wavelength λ ≈ 633 nm, ALV GmbH, Langen, Germany), located at the PSCM Grenoble. Script-based automated measurements enable the investigation of the sample if the kinetic timescales of the sample evolution are significantly longer than the time needed for a scan of all scattering angles. The sample temperature was kept at 20°C using a water bath. At each angle, 30 s measurements were acquired five times. Measurements were performed iteratively, without waiting time, resulting in a kinetic time resolution of half an hour. The individual correlation functions were normalized, dust-induced outliers were removed (less than 1% of all data), and the individual correlation functions were subsequently averaged before further analysis.

### 2.5. Small-angle neutron scattering

Data were acquired on the small-angle scattering instrument D33 at the Institut Laue–Langevin (ILL, Grenoble, France) (Dewhurst *et al.*, 2016 ▸) at room temperature during the beamtime exp 8-04-953 (Mateo Miñarro *et al.*, 2023 ▸). Samples were prepared in Eppendorf vials and subsequently transferred to quartz cuvettes. The sample-to-detector distances were 13 m for the rear detector and 1.7 m for the front detector with a collimation of 12.8 m. The neutron wavelength used was 4.65 Å. These settings allowed a *q* range from 0.004 to 0.440 Å^−1^ to be covered. The sample environment was temperature-controlled at 295 K.

Prior to further analysis, data were corrected by their respective transmissions. Corrections for electronic background noise were done by measuring ^10^B_4_C. The scattering of the empty cell was subtracted. Data were calibrated to absolute intensity via normalization to attenuated empty beam measurements. 2D data were radially averaged using the program *Grasp* (Dewhurst, 2023 ▸), exported as *I* versus *q* curves and further analyzed with custom Python scripts.

### 2.6. Neutron spin echo

Spin echo data were acquired on the WASP spectrometer at the ILL in Grenoble. AlMgSi alloy (EN AW-6060) double-walled cylindrical sample holders with a 0.3 mm gap and a 15 mm outer diameter were used. These holders were designed to have a low neutron scattering background. The temperature was stabilized at 298 K using a cryostat mounted on the spectrometer. Measurements were performed with an acquisition time of approximately two hours per spectrum with a wavelength λ = 7 Å.

Additional spin echo data were recorded on the IN11A spectrometer at the ILL in Grenoble. The samples were filled into quartz cuvettes and maintained at room temperature in a box-shaped sample environment suitable for small scattering angles. Measurements were performed using wavelength λ = 8 Å at 2θ (°) = 3.7, 5.5, 7.3, 9.5, 11.2, 14.5 corresponding to scattering vector magnitudes *q* (Å) = 0.05, 0.075, 0.1, 0.13, 0.134, 0.198.

The resolution functions of the instrument were determined for each experimental setup using the elastic scattering of graphite. The resulting intermediate scattering functions *I*(*q*, τ), depending on the scattering vector magnitude *q* and the Fourier time τ, were corrected for the buffer background dynamics. Further data analysis was performed using custom Python scripts.

### 2.7. Neutron backscattering

Time-resolved neutron backscattering experiments were performed on the neutron backscattering spectrometer IN16B (Frick *et al.*, 2010 ▸) using its phase space transformation chopper (Hennig *et al.*, 2011 ▸) and Doppler-driven monochromator, with Si(111) monochromator and analyzer crystals, achieving an energy resolution Δ*E* ≈ 0.9 µeV FWHM (corresponding to an observation or coherence time of ∼4 ns) at 2.08 meV incident neutron energy. The samples were prepared in Eppendorf vials and transferred to double-walled cylindrical aluminium sample holders made from the same Al alloy as the WASP sample holders, but with a 22 mm outer diameter and a 0.15 mm gap. The temperature was stabilized at 298 K using an Orange cryostat mounted on the spectrometer. Data were reduced using *Mantid* (Arnold *et al.*, 2014 ▸; Akeroyd *et al.*, 2013 ▸) and further analyzed using custom Python scripts. Diffusive dynamics result in quasi-elastic neutron scattering (QENS) in the energy domain accessed by neutron backscattering. This scattering was sampled by elastic and inelastic fixed window scans (FWSs) (Beck *et al.*, 2024 ▸; Frick *et al.*, 2012 ▸) as well as by full QENS spectra in an iterative way, allowing us to follow the kinetic process with a good kinetic time resolution with 13 min repetition rate. We note that IN16B and D33 measure the total, *i.e.* the sum of nuclear spin-incoherent and coherent, scattering, whereas WASP can separate these signals via neutron spin polarization analysis (see Section 3.5). For data collected on IN16B, we separated the D_2_O solvent contribution in the data analysis, according to a measurement of a pure D_2_O reference sample. In the *q* range covered by IN16B (0.2 ≤ *q* ≤ 1.9 Å^−1^), there are no further structural features in the simultaneously recorded diffraction patterns [see Fig. 3(*e*)]. For this reason and due to the dominant scattering from the ^1^H atoms of the proteins, we assumed that this part of the scattering function was incoherent.

## 3. Results and discussion

### 3.1. Description of the kinetic crystallization process

**Figure 1:**
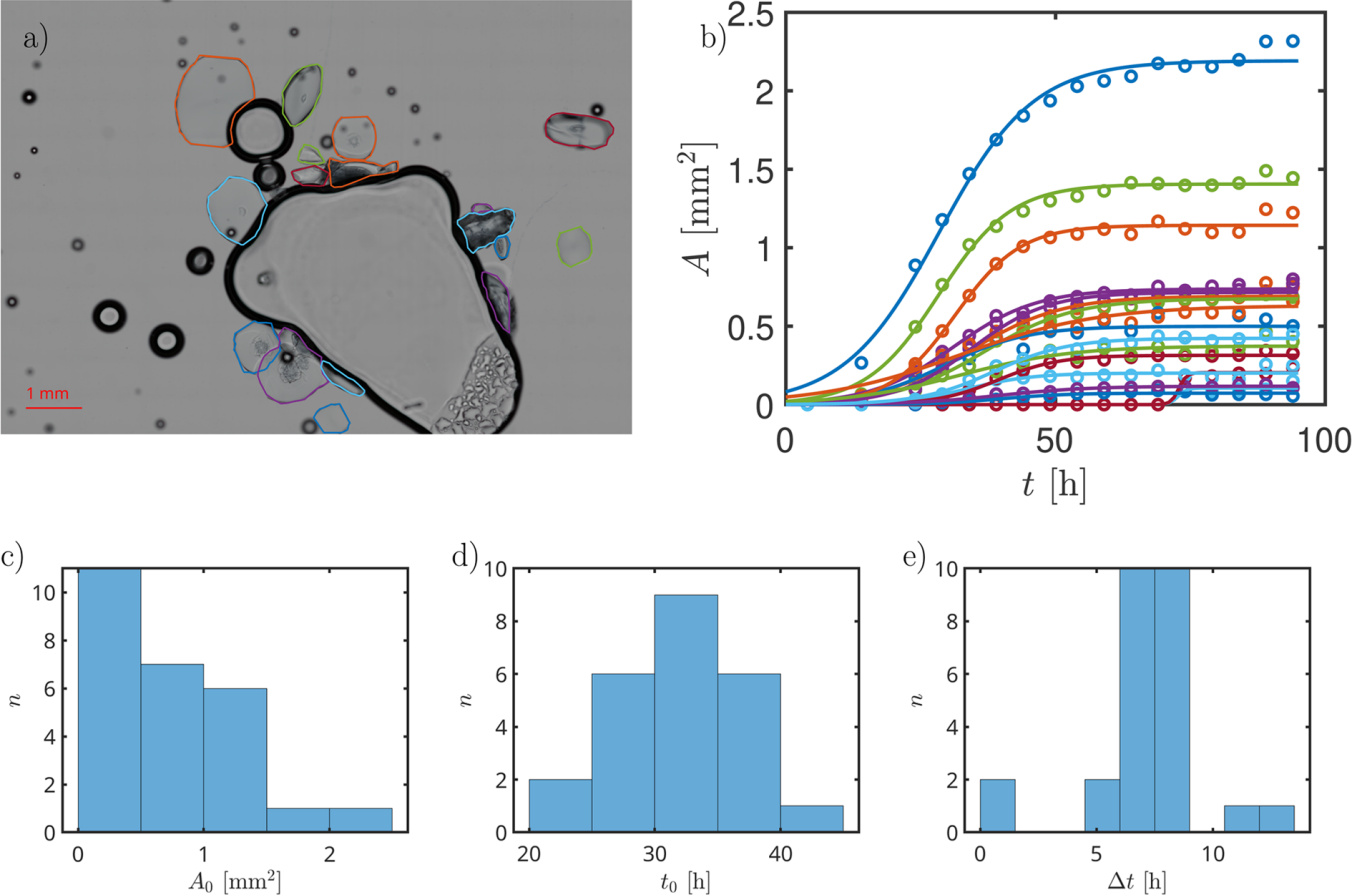
(*a*) Time-dependent microscopy images for *c*_p_ = 75 mg ml^−1^ with *c*_s_ = 4.5 m*M* LaCl_3_ in D_2_O analyzed by determining the 2D-projected area of crystallites through their borders. (*b*) The time-dependent area *A*(*t*) approximated for each crystal individually using equation (2) [*A*(*t*) = *f*(*t*) in equation (2)] to obtain the time of fastest crystal growth *t*_0_, the maximum area *A*_0_ = *f*_0_ and Δ*t* defining the growth rate of the crystals. By investigating several crystals, histograms of these observables can be created [(*c*), (*d*), (*e*)].

**Figure 2:**
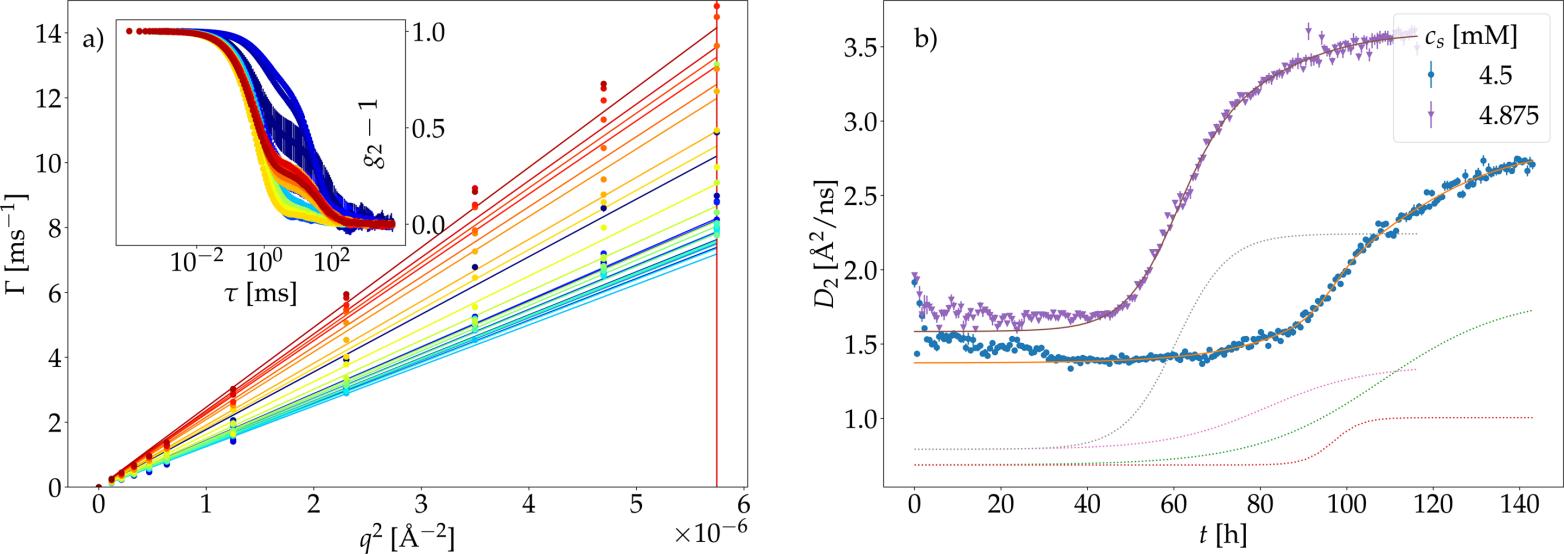
(*a*) Time-dependent results from the DLS measurements on HSA *c*_p_ = 75 mg ml^−1^ with *c*_s_ = 4.5 m*M* LaCl_3_ in D_2_O. The inset of (*a*) displays example DLS correlation functions acquired at different times during the crystallization, fitted by equation (3), allowing for the sum of two decays. The main part of (*a*) shows the *q* dependence of the decay rates (symbols) for the faster decay Γ_2_ of these two fitted contributions. The time dependence is color coded (blue to red) showing each 10th data set. The decay rates are fitted by 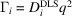 (solid lines). (*b*) The kinetic evolution of the fast diffusion coefficients *D*_2_ from DLS, attributed to the protein monomers, for the two samples investigated (symbols), described by the sum of two sigmoid functions, equation (2) (solid lines). The individual sigmoid contributions are represented by dotted lines.

We assume that for classical crystallization processes the time evolution of the crystal concentration can be described by a sigmoid function, also denoted logistic function (Nanev & Tonchev, 2015 ▸; Nanev, 2017 ▸): 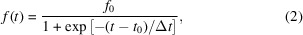
where *f*_0_ is the maximum value, *t*_0_ is the time at which the crystal is growing the fastest and Δ*t* together with *f*_0_ defines the fastest growth rate, *f*′(*t* = *t*_0_) = *f*_0_/(4Δ*t*) at *t*_0_. If the process contains intermediate steps, a combination of sigmoid functions can be used. We find that equation (2) describes the kinetic evolution of crystallization observed with all experimental methods of this work, as shown later in Figs. 1(*b*), 2(*b*), 4, 5 and 7.

### 3.2. Optical microscopy

Optical microscopy images were collected to visually follow the time dependence of the crystal growth. For different timesteps, the shapes of the crystals have been identified, allowing us to determine the time-dependent 2D-projected crystal area. Videos of the time-dependent microscopy images showing the crystal growth and the identified crystal boundaries are available in the supporting information.

Fig. 1 ▸(*b*) shows the time dependence of the crystal size in the 2D projection observed by transmission microscopy for HSA *c*_p_ = 75 mg ml^−1^ with *c*_LaCl_3__ = 4.5 m*M* in D_2_O. It can be observed that the different crystals follow a time dependence which can be reasonably well described by the scaled and shifted sigmoid function given by equation (2).

As visible in Fig. 1 ▸(*c*), the size distribution *A*_0_(*n*) of the *n* different crystals is dominated by small crystals. The maximum crystal growth rate can be described by a bell-shaped distribution centered around *t*_0_ ≈ 32 h [Fig. 1 ▸(*d*)]. The characteristic time Δ*t* is around 7 h for most of the observed crystals [Fig. 1 ▸(*e*)]. The error-weighted means are given in Table 1 ▸ for the two different sample conditions. In addition to the time dependencies, the investigation of the microscopy images indicated a preference of the crystals to grow at the interface between the air bubbles located within the observation area and the solution. This observation is in agreement with previous studies of the same system in H_2_O (Banks *et al.*, 2024 ▸). Therefore, there does not appear to be a significant isotope effect.

Similar results have been observed at slightly higher salt concentration (*c*_LaCl_3__ = 4.875 m*M*; see supporting information). However, the lower number of observed crystals in the observation field results in less smooth histograms. While Δ*t* and *t*_0_ seem to be normally distributed in the case of *c*_s_ = 4.5 m*M* [Figs. 1 ▸(*d*) and 1 ▸(*e*)], the distributions for *c*_s_ = 4.875 m*M* seem to deviate from normal distributions.

### 3.3. Small-angle neutron scattering

Small-angle scattering measurements enable the characterization of crystallization pathways and can distinguish between crystal precursors and protein clusters (Maier *et al.*, 2021 ▸; Sauter *et al.*, 2015*b* ▸). Here, we use SANS to determine the Bragg peak position depending on the sample conditions by systematically changing the salt and protein concentrations. As shown in Fig. S2, the Bragg peak position and therefore the underlying crystal structure significantly depend on the salt concentration in the initial solution. This Bragg peak, for HSA with *c*_s_ = 4.875 m*M* located at *q* = 0.15 Å^−1^ corresponding to a lattice spacing of *d* = 2π/*q* = 4.18 nm, can be tentatively attributed to the protein–protein nearest neighbor distance in a dense packing including hydration water, given the HSA hydrodynamic radius (Maier *et al.*, 2020 ▸; Akbarzadehlaleh *et al.*, 2020 ▸). To investigate the origins influencing the different pathways resulting in the distinct structures, we follow the crystallization processes with kinetic measurements applying different techniques.

### 3.4. Dynamic light scattering

To probe the long-time collective diffusion, the samples were examined using time-dependent dynamic light scattering. The correlation functions obtained feature a clear shoulder [inset Fig. 2 ▸(*a*)]. A double exponential decay was therefore used to describe the normalized correlation function *g*_2_ − 1 for all *q* values studied: 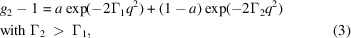
taking into account the contributions of small crystals or precursors with a decay rate Γ_1_ and dissolved proteins in solution with a decay rate Γ_2_. The fit parameter 0 ≤ *a* ≤ 1 defines the ratio of the two contributions. The *q* dependence of the decay rates Γ_1,2_ is described by 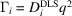 to determine the long-time translational collective diffusion coefficient 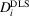 for the clusters and monomers in solution [Fig. 2 ▸(*a*)]. A direct conversion into a hydrodynamic radius is not possible because several factors, such as the high protein concentration (Pan *et al.*, 1995 ▸) and the presence of the multivalent salt (Soraruf *et al.*, 2014 ▸), influence the diffusion coefficient. We therefore focus on the interpretation of the relative changes of the observed diffusion coefficients.

Fig. 2 ▸(*b*) displays the time dependence of 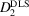 for both HSA samples investigated. In both cases, an increase of the diffusion coefficient that describes the dissolved proteins in solution can be observed. This effect can be explained by an inverse crowding effect that results in an effective reduction of protein concentration in the solution during crystal growth, as previously observed (Beck *et al.*, 2019*b* ▸). Simultaneously, the diffusion coefficients describing the precursors and small crystals decrease with time. The time dependence of the diffusion coefficients of the clusters is shown in Fig. S5.

Since the samples showed slight turbidity at the beginning of the measurements, the first measurement points might be influenced by multiple scattering effects. These effects might exist in the homogeneous solution due to the high protein concentration. However, since the overall concentration is constant in time, the relative changes in the observables are due to changes in the samples. The good kinetic time resolution of the DLS measurements in combination with the overall long measurement time allows for a disentanglement of two different processes over time. Multistep crystallization processes have been observed previously in salt-induced protein crystallization processes (Maier *et al.*, 2021 ▸; Alexandrov & Makoveeva, 2023 ▸; Sauter *et al.*, 2016 ▸). The sum of two sigmoidal functions has therefore been used for a description of the time dependence. The characteristic times Δ*t* and *t*_0_ for the fits shown in Fig. 2 ▸(*b*) are listed in Table 1 ▸.

### 3.5. Neutron spin echo spectroscopy

**Figure 3:**
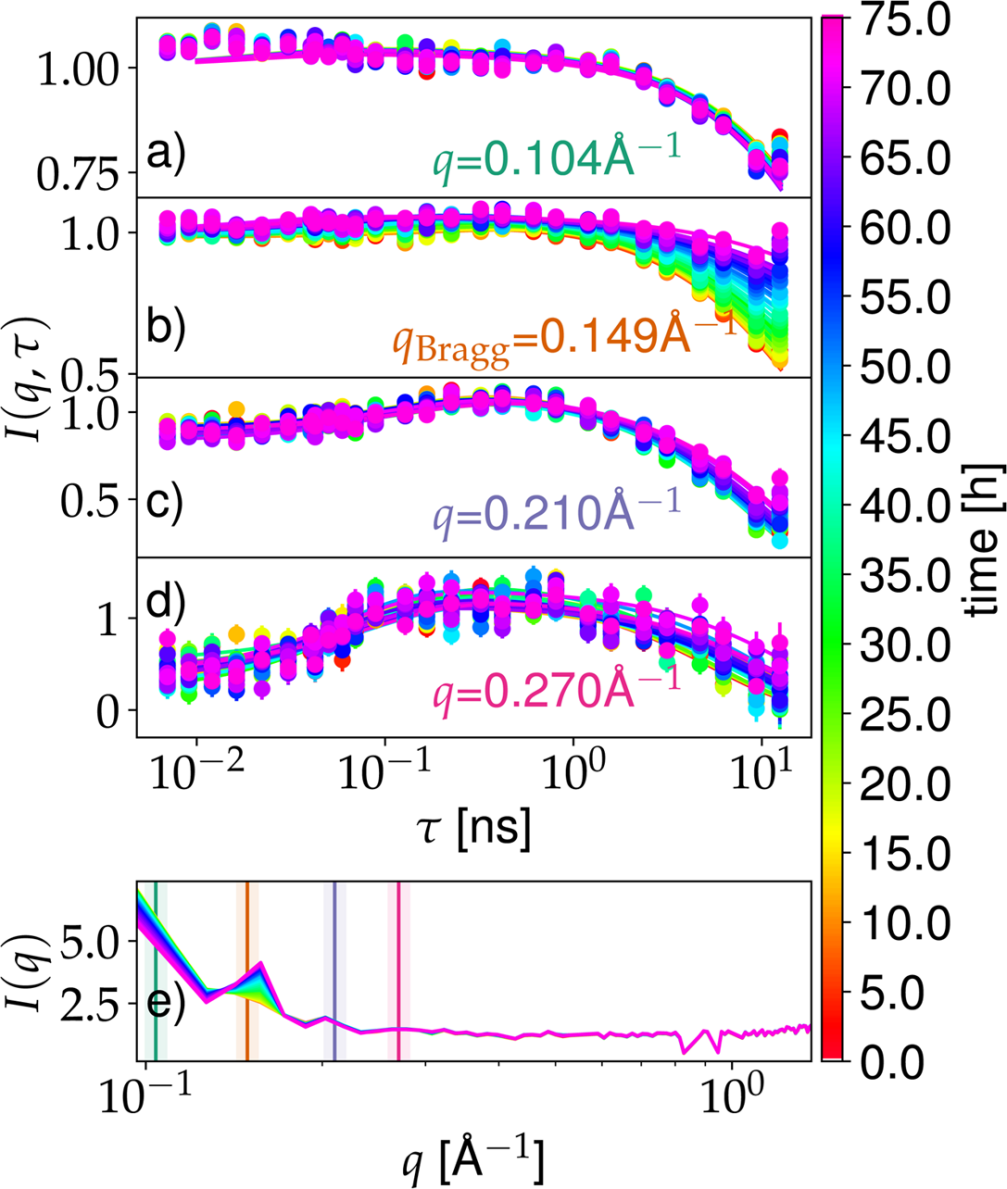
Time-dependent neutron spin echo spectroscopy measurements (symbols) acquired on WASP for different *q* values as indicated in the corresponding subplots for *c*_s_ = 4.875 m*M*. Circles and lines indicate measured points and fits with corresponding models described in the text, respectively. The age of the sample, representing the duration since the initiation of the crystallization process, is indicated by the color gradient displayed on the right. The lowest subplot displays the kinetic diffraction data versus *q*, nicely showing the growth of the Bragg peak over time. Solid lines and the shaded areas indicate the measured *q* ranges for the intermediate scattering functions and the *q* resolution assuming Δλ/λ = 8%.

To observe diffusion on the length scale of the individual proteins, we investigated the crystallization process with kinetically time-resolved NSE spectroscopy, allowing access in the short-time limit to collective and self-diffusion on the ångström level. Different behaviors could be observed in the time dependence of the intermediate scattering functions. The intermediate scattering functions displayed in Figs. 3 ▸(*a*)–3 ▸(*d*) are given by 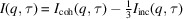, where *I*_coh_ and *I*_inc_ are the coherent and incoherent contributions, respectively (Hoffmann, 2021 ▸). For *q* < 0.15 Å^−1^, the coherent decay *I*_coh_(*q*, τ) dominates *I*(*q*, τ) [Figs. 3 ▸(*a*) and 3 ▸(*b*)], shown by the monotonic drop of *I*(*q*, τ) with τ. In contrast, for *q* ≥ 0.21 Å^−1^, *I*(*q*, τ) shows an initial increase with τ for τ < 0.2 ns [Figs. 3 ▸(*c*) and 3 ▸(*d*)], which is the signature of an incoherent decay on subnanosecond timescales. Therefore, both the coherent and incoherent contributions of the scattering function have to be modeled. Since it cannot be ruled out that the intermediate scattering functions have already decayed before the first Fourier time τ measured, the two different scaling parameters for the coherent (*a*_coh_) and incoherent (*a*_inc_) contributions cannot be fixed according to theory (Hoffmann, 2021 ▸). We fix the sum of the two parameters to be equal to the minimal value of the measured Fourier times with τ < 15 ps. The intermediate scattering function can therefore be described as 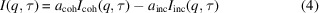
with a fixed parameter *a*_coh_ − *a*_inc_. Given the maximum observation time of τ_max_ ≈ 10 ns, a disentanglement of the internal and global dynamics of the proteins as in other studies (Biehl & Richter, 2014 ▸; Haris *et al.*, 2022 ▸; Buvalaia *et al.*, 2023 ▸; Sohmen *et al.*, 2023 ▸) cannot be performed. Assuming that the internal dynamics of the proteins are independent of their local environment, time-dependent changes in the observed mean diffusion coefficient can be assigned to changes in the apparent center-of-mass diffusion of the proteins. At high *q* values above *q* = 0.3 Å^−1^, the scattering signal is dominated by incoherent scattering.

At the Bragg peak positions *q*_Bragg_, the intermediate scattering function is a combination of the scattering of the free proteins in solution and the crystals. The coherent part of the scattering function *I*_coh_(*q*_Bragg_, τ) in equation (4) can therefore be written as 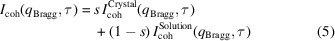
with the scalar s being linked to the fraction of proteins in the crystals (Beck *et al.*, 2019*b* ▸). Since the crystals can be assumed as immobile on the observed timescale, the intermediate scattering function simplifies to unity, *i.e.*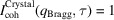. The intermediate scattering function describing the proteins in solution at *q*_Bragg_ was fixed on the basis of the adjacent *q* values assuming Fickian diffusion. In Fig. 3 ▸, the fit results of the kinetic intermediate scattering function are displayed for different momentum transfers ℏ*q* in the first four subplots. While for *q* = 0.149 Å^−1^ the intermediate scattering function is modeled using *s* as a free parameter in equation (5) which is inserted in equation (4), the other *q* values investigated have no Bragg peaks and are therefore analyzed with *s* = 0. For the different contributions to the intermediate scattering function, exponential decays are used, 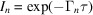 with *n* representing the coherent and incoherent contributions. The lower part of Fig. 3 ▸ depicts the kinetic diffraction data acquired simultaneously on WASP. Vertical lines indicate the *q* values at which the intermediate scattering functions shown above are acquired. The same color code indicating the age of the sample applies to all plots.

**Figure 4:**
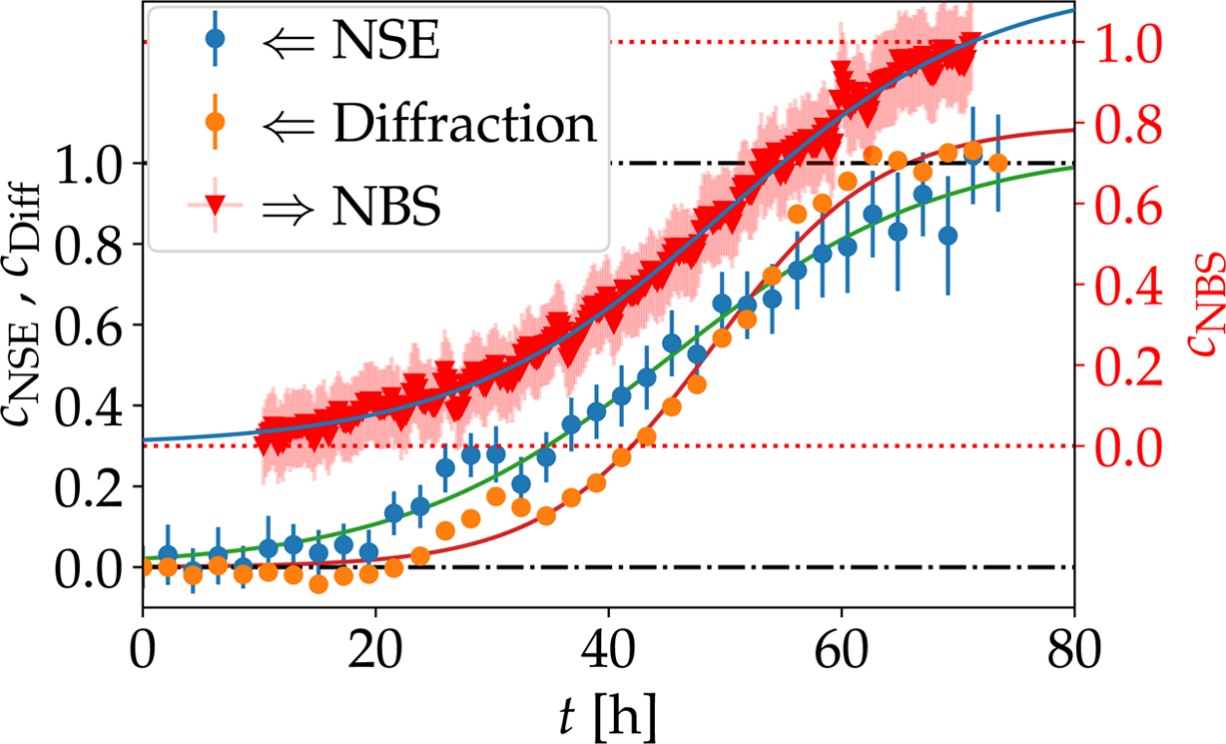
Crystalline fraction *c*_NSE_ of the HSA solution sample with 4.875 m*M* LaCl_3_ and *c*_p_ = 75 mg ml^−1^ obtained from the diffusive properties determined by NSE (blue symbols), and *c*_Diff_ from the structural properties determined by *in situ* SANS recorded simultaneously with NSE (orange symbols), as well as crystalline fraction *c*_NBS_ obtained from the diffusive properties determined by NBS on an identical sample (red symbols, right-hand side *y* axis). The kinetic trend of the elastic contribution obtained from the NSE (*c*_NSE_, blue symbols) measurements, determined from the parameter *s* in equation (5), agrees well with that from NBS (*c*_NBS_, red). The NBS data set is shifted by 10 h. The crystal fraction determined by the diffraction data (*c*_Diff_, orange) increases later than the elastic contribution from NSE and NBS, indicating the presence of non-crystalline particles before the crystal formation. All time dependencies (solid lines) have been fitted by equation (2). All data have been background-subtracted and normalized such that the maximum data value becomes 1 (indicated by black dashed–dotted and red dotted lines for data captured on WASP and IN16B, respectively). A free scaling parameter was incorporated into the fitting procedure to compensate for incomplete capture of the crystallization process.

The collected diffraction data are characterized by a growing Bragg peak for the sample with *c*_s_ = 4.875 m*M* LaCl_3_. At each timestep, we determined the contribution of the crystal to the scattering signal. We estimated the scattering from the dissolved protein at *q*_Bragg_, *i.e.* the signal in the absence of Bragg scattering, by interpolation from the values of the scattering function measured at *q* ≠ *q*_Bragg_. We subtracted this interpolated value from the measured scattering signal including the Bragg peak, thus resulting in the crystal signal. By assuming the sample to be homogeneous without crystals in the initial state and fully crystallized in the last measurement, the crystal fraction in the solution can be estimated by subtracting the value at *t* = 0 and normalizing the result by *t*_final_. The time dependence of the crystal growth is illustrated in Fig. 4 ▸ and shows a good agreement with the crystal fraction determined from the intermediate scattering function.

**Figure 5:**
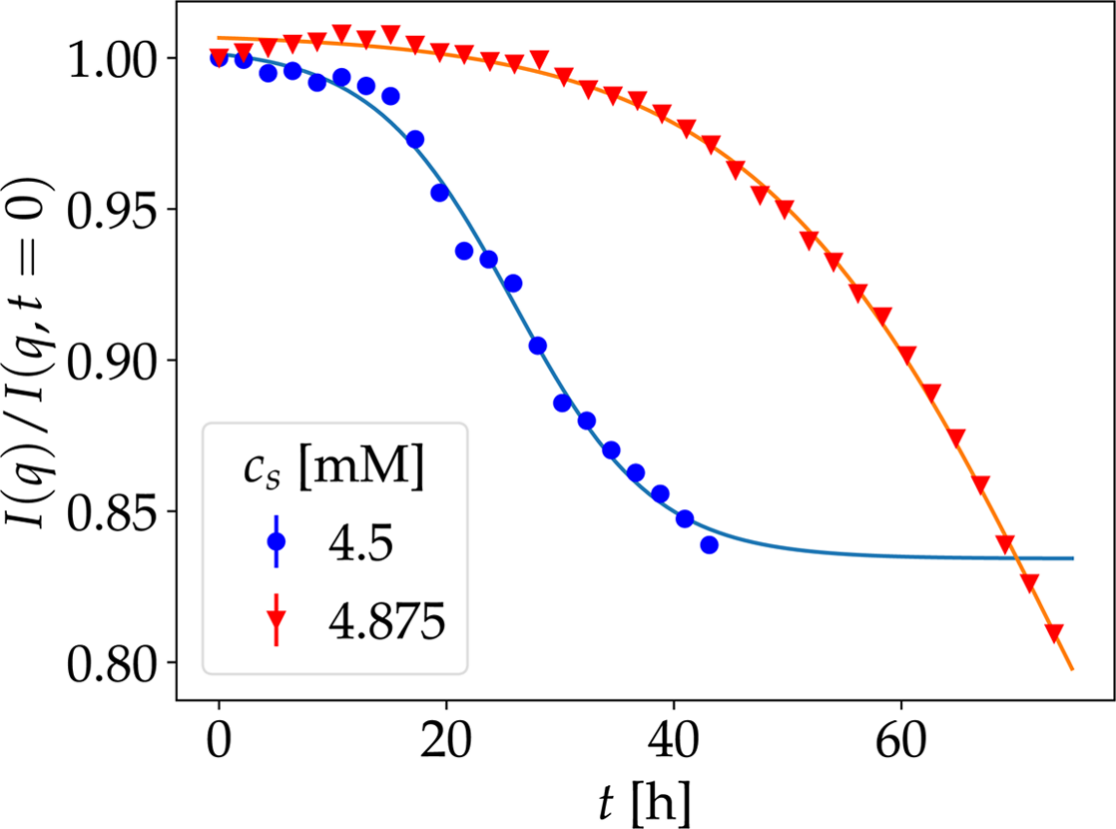
Normalized time-dependent diffraction signal intensity at *q* = 0.096 Å^−1^ recorded simultaneously with the neutron spin echo measurements on WASP during the crystallization process of HSA [*c*_p_ = 75 mg ml^−1^ in the presence of LaCl_3_ for *c*_p_ = 4.5 m*M* (blue filled circles) and *c*_s_ = 4.875 m*M* (red filled triangles)]. Solid lines are fits of equation (2) including a constant background. Fit results are given in Table 1 ▸.

In addition to the analysis of the regions around the Bragg peaks, the diffraction at low *q* can also be investigated, where coherent scattering dominates. Previous SANS studies have shown the suitability of kinetic SANS measurements to investigate the kinetic properties (Maier *et al.*, 2021 ▸; Sauter *et al.*, 2016 ▸). The time dependence of the lowest momentum transfer of the diffraction data, *q* = 0.096 Å^−1^, recorded simultaneously during the WASP measurements [Fig. 3 ▸(*e*)], has been fitted using equation (2) with an additional background. The advantage of this approach is that it is independent of the presence of Bragg peaks in the observed *q* range. Therefore, it is possible to extract the characteristic times of the crystallization process for both sample conditions (Fig. 5 ▸) by investigating *I*(*q*, *t*)/*I*(*q*, *t* = 0). However, the relatively low *q* resolution does not allow the separation of different contributions as done in previous SANS studies (Sauter *et al.*, 2016 ▸; Maier *et al.*, 2021 ▸).

The time dependence of the crystal fraction of the HSA sample with 4.875 m*M* LaCl_3_ determined from diffraction on WASP, showing a Bragg peak in the observed *q* range, can also be described with equation (2) (orange points in Fig. 4 ▸), with the fit parameters *t*_0_ = (48.98 ± 0.63) h and Δ*t* = (7.15 ± 0.45) h. The parameters determined from the scattering data represent an ensemble average of the entire system and therefore have to be compared with the distributions of the parameters determined from microscopy. Differences between the techniques might be due to specific sample containers (quartz glass for microscopy, cylindrical aluminium sample holders for NSE measurements) and other control parameters such as sample temperature. The non-monotonic time dependence at around 30 h in the data set recorded on WASP might be due to crystals falling out of the observed sample area.

### 3.6. Neutron backscattering spectroscopy

**Figure 6:**
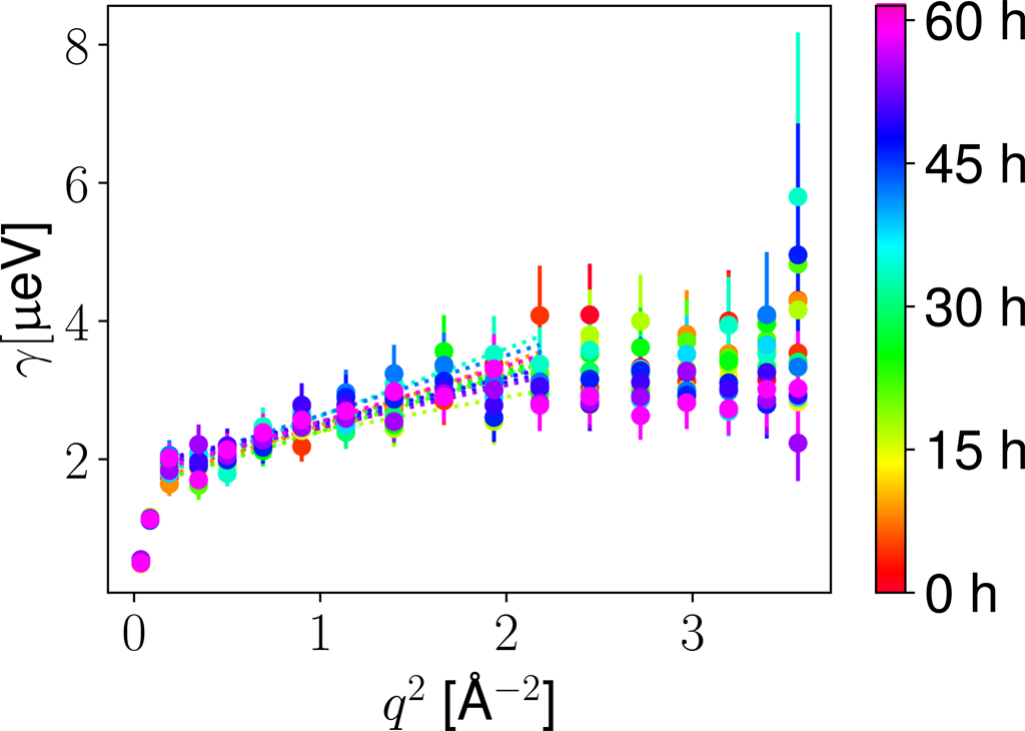
*q* dependence of Lorentzian linewidth γ on the HSA solution sample with *c*_s_ = 4.875 m*M* LaCl_3_ and *c*_p_ = 75 mg ml^−1^ determined from the neutron backscattering fixed window scans by the ratio method using the energy offsets 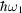 = 1 µeV and 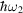 = 3 µeV (see main text). Different timesteps are color coded. The *q* dependence has been fitted using equation (6) (dotted lines). For better visibility, each 20th step is shown.

**Figure 7:**
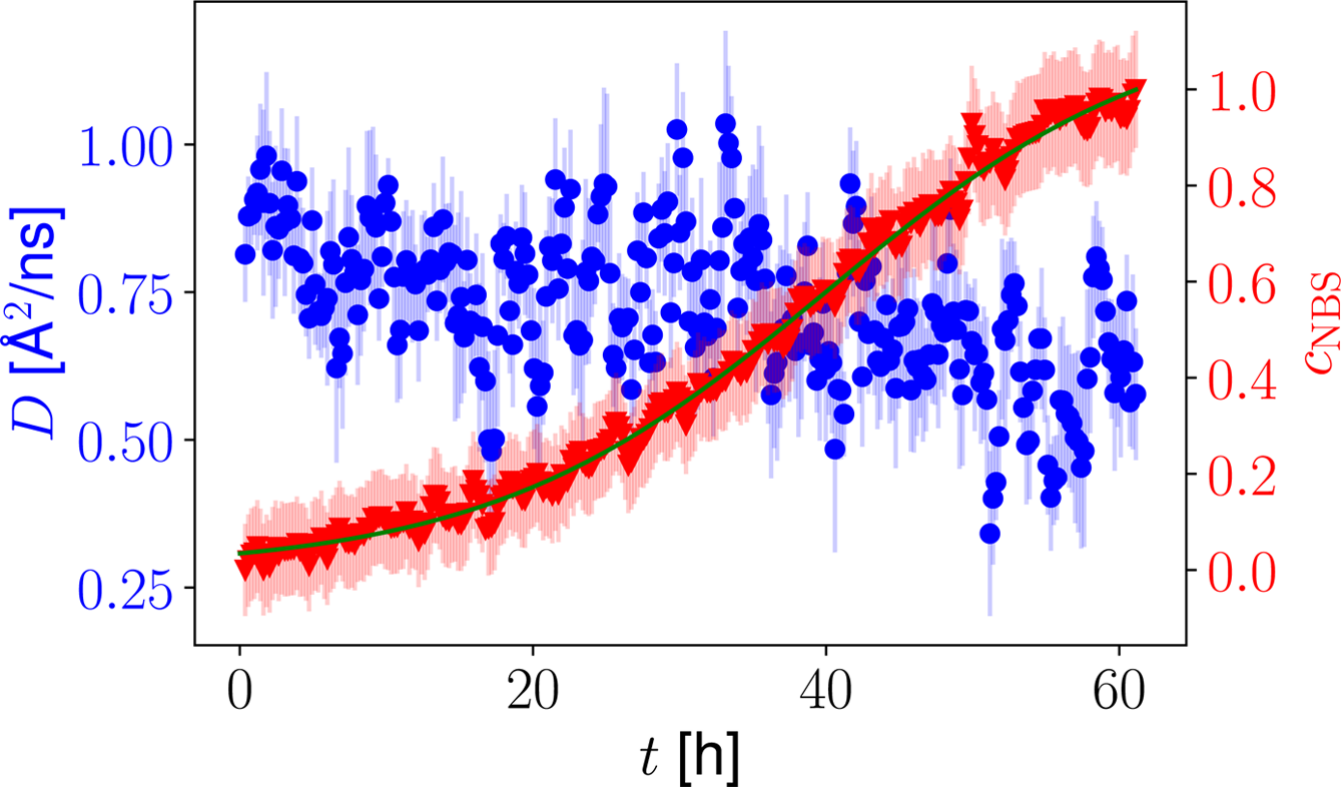
Averaged diffusion coefficient *D* of the dissolved proteins obtained from the ratio analysis, equation (6) (blue circles, left axis), and the fraction of immobile proteins *c*_NBS_ [equation (8), red triangles, right axis] as a function of time determined from FWSs at 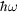 = 0, 1 and 3 µeV, for the HSA solution sample with *c*_s_ = 4.875 m*M* LaCl_3_ and *c*_p_ = 75 mg ml^−1^. While the increasing fraction of immobile proteins *c*_NBS_ illustrates the protein crystallization process, which has been fitted by equation (2), the diffusion coefficients *D* show only a small decrease with time.

IN16B detects the total scattering (see Section 2.7). Due to the absence of spin polarization analysis, an unambiguous separation of the coherent and incoherent scattering cannot be performed (Sarter *et al.*, 2024 ▸; Nidriche *et al.*, 2024 ▸; Gaspar *et al.*, 2010 ▸; Arbe *et al.*, 2020 ▸). Nevertheless, from the flat diffraction signal for *q* > 0.2 Å^−1^ [see Fig. 3 ▸(*e*)], we can assume that our signal recorded in NBS subsequent to solvent subtraction is dominated by the incoherent scattering of the ^1^H atoms of the proteins. To determine the short-time self-diffusion of the proteins in solution, time-dependent FWS data have been analyzed via the ratio analysis established previously (Beck *et al.*, 2024 ▸) using the energy offsets 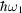 = 1 µeV and 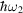 = 3 µeV. Since both energy transfers are close to but clearly outside of the instrumental resolution function, the FWS ratio mainly arises from the apparent center-of-mass diffusion *D* of the proteins in solution. The ratio of the intensities at these two offsets can, thus, be used to obtain a Lorentzian linewidth γ, based on assumptions for the entire scattering function (Beck *et al.*, 2024 ▸). Fig. 6 ▸ displays the *q* dependence of γ for different timesteps during the crystallization process. This *q* dependence of γ has been described for each timestep by 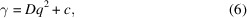
where *D* is an effective short-time diffusion coefficient that approximates the center-of-mass diffusion within the limitations of the FWS ratio analysis method. This effective diffusion coefficient might contain an additional contribution from the internal diffusion of the protein and might average over the center-of-mass diffusion coefficients of differently sized clusters. The offset *c* in the equation (6) above[Disp-formula fd6], in part accounting for strongly localized or jump-like internal motions, also results from limitations of the FWS analysis. Nevertheless, as shown previously, the small energy offsets favor the probing of the apparent center-of-mass diffusion, which is a combination of translational and rotational diffusion (Grimaldo *et al.*, 2015*c* ▸), and result in quantitative agreement in the case of high protein concentrations (Beck *et al.*, 2024 ▸). Previous studies have also shown that the internal diffusive processes on the observed time and length scale are only slightly influenced by the formation of clusters (Beck *et al.*, 2021 ▸; Beck *et al.*, 2018 ▸). Importantly, relative changes in the kinetic time dependence of the diffusion coefficients can therefore be related to changes in the apparent center-of-mass diffusion. The averaged diffusion coefficient obtained from a fit in the range 0.4 < *q* < 1.4 Å^−1^ is shown in Fig. 7 ▸. The effective diffusion coefficient *D* is characterized by a slight slowing down over time, which may be explained by the vanishing contribution of the initially free monomers which become part of crystals. The relative concentration of the remaining clusters in the solution increases and therefore their contribution to the averaged diffusion coefficient *D* is stronger. Since *D* is influenced by the volume fraction (Roosen-Runge *et al.*, 2011 ▸; Grimaldo *et al.*, 2014 ▸), the composition of the sample (Beck *et al.*, 2022 ▸; Grimaldo *et al.*, 2019 ▸) and the salt concentration (Grimaldo *et al.*, 2015*b* ▸; Beck *et al.*, 2021 ▸), which might vary during the crystallization process, a quantitative separation is not possible without further assumptions.

Subsequently to the determination of the effective diffusion coefficient *D*, the contribution of the diffusing particles *S*_dif_ to the scattering function at 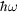 = 0 µeV was extrapolated using both inelastic FWSs and calculated γ. The scaling parameter *s*_FWS_ is therefore determined such that the Lorentzian function 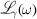 matches both inelastic FWSs: 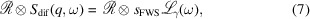
where 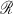 is the resolution function. Considering also the elastic FWS, it is then possible to determine the elastic contribution *c*_e_ in the scattering signal as a function of time, describing the incoherent scattering function as 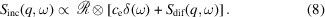
The sample container only contributes to the elastic scattering, *i.e.* to the term *c*_e_δ(ω) in equation (8). To not increase the errors, we did not subtract the container signal from the raw data. Instead, we subtracted the value *c*_e_(*t* = 0) as a constant contribution from all data points and normalized to the value at the final time, *c*_e_(*t* = *t*_max_). For each timestep, the elastic fraction *c*_e_ was averaged over 1.1 < *q* < 1.8 Å^−1^. The kinetic time dependence was normalized to the interval [0, 1] to remove the contribution of the container to obtain the crystal fraction *c*_NBS_. The presence of immobile proteins during the initial acquisitions and an incomplete observation of the kinetic process might influence the overall absolute values of *c*_NBS_. The kinetic time constants, however, are not influenced by such a normalization, which allows a better comparison with other techniques. Its time dependence is shown in Fig. 7 ▸. While the averaged short-time self-diffusion coefficient is not significantly influenced during the crystallization process, the contribution to the elastic scattering clearly indicates the crystal growth over time. A description of the time dependence using equation (2) results in *t*_0_ = (40.0 ± 0.35) h and Δ*t* = (11.48 ± 0.16) h.

As indicated previously, the investigated system also crystallizes at interfaces. Changes in the sample environment can therefore significantly change the onset of the crystallization process. The kinetic time dependence Δ*t* of the crystal fraction obtained from NBS is in good agreement with the kinetic dependence determined with NSE (Fig. 4 ▸) if the data are shifted 10 h, influencing only *t*_0_. The properties of the interface therefore seem to mainly influence the nucleation time of the system. Once crystal seeds are present, the kinetic process appears to follows the same pathway.

### 3.7. Interpretation of the time dependencies found across the different methods

By combining results from different techniques, probing different diffusive processes on different time and length scales, it is possible to obtain deeper insights into crystallization pathways characterizing the systems. Importantly, the scattering techniques (DLS, NSE, NBS, diffraction) probe the ensemble average, while time-resolved microscopy accesses individual protein crystals over time.

The spectroscopy techniques separate the monomer and crystallite signals dynamically, since the motion of proteins within the crystallites is reduced compared with the free monomer diffusion. Moreover, due to high momentum transfers, NBS probes length scales smaller than the protein diameter. For our samples, this corresponds to the incoherent limit where the signal is proportional to the scattering cross-section, such that coexisting protein monomers and crystallites contribute equally to the signal in terms of protein number density.

Therefore, the spectroscopy techniques provide information on coexisting protein monomers during the formation of the crystallite. NSE and NBS probe the collective and self-diffusion, respectively, in a short-time limit on the nanosecond timescale, while DLS accesses the collective long-time diffusion on a millisecond scale.

For the HSA–LaCl_3_ system, the results for the kinetic evolution of the crystallization from all techniques can be described by a sigmoid function [equation (2)]. This evolves with similar characteristic times [on the order of 40–80 h for *t*_0_ and 5–14 h for Δ*t*] on all observation scales, as summarized in Table 1 ▸, with small but possibly systematic differences as discussed further below. We note that microscopy observes crystallite face areas. The associated crystallite volumes can be estimated by assuming cubic crystallites (see supporting information for details). In Table 1 ▸, the additional line labeled ‘Microscopy corr.’ reports the fit parameters for the thus-converted microscopy results. In view of the observation scales of the techniques employed, the results in Table 1 ▸ suggest that the diffusion on a multiscale level (long-time collective and short-time self and collective) and the formation of the crystallite structure evolve in parallel and not sequentially.

Considering the above observation of congruent evolution on all observation scales as the big picture, we now discuss the comparatively small differences. In contrast to the other techniques, the time dependence of the DLS data requires two sigmoid functions for a good description [Fig. 2 ▸(*b*)]. However, this second sigmoid to describe the DLS results only becomes significant for kinetic evolution over very long times. These long times were not reached by the neutron experiments due to the limited availability of neutron beamtime. Therefore, the shorter total duration of the neutron experiments presumably explains the absence of the second kinetic process in the neutron data. Additional reasons may arise from the different observation timescales (nanoseconds in the case of NSE and NBS; milliseconds in the case of DLS), from the sparseness of the data set or from the experimental errors, which might result in a sufficient description of the kinetic process using only one sigmoid function for neutron data. Restricting the time range in the DLS data set to *t* ≤ 80 h and describing the data with one single sigmoid function results in an incomplete description of the time dependence (see supporting information).

In addition to *t*_0_ [equation (2)], the crystallization process is also characterized by the overall kinetics. The speed is here given by the parameter Δ*t* [equation (2)]. By comparing this parameter for the different techniques used to investigate the diffusive properties (NSE, NBS, DLS), a good agreement between short-time diffusion and long-time diffusion behavior can be observed for the sample with *c*_s_ = 4.875 m*M* (Table 1 ▸). This agreement might suggest that the same underlying kinetic process dominates on all length scales. We observe a difference in the kinetic evolution between diffraction results [Fig.  3 ▸(*e*)] (*i.e.* structural information collected *in situ* during the NSE measurements, in particular at low momentum transfers *q*) and the NSE measurements themselves [Figs. 3 ▸(*a*)–3 ▸(*d*)]. This difference becomes apparent in the plot versus time (Fig. 4 ▸). The diffusion from DLS, NSE and NBS [Figs. 2 ▸(*b*) and 4 ▸] might indicate structural rearrangements in the protein assemblies, which emerge as disordered aggregates already sufficiently large to appear as elastic contributions and gradually transform into crystals. This improvement of crystallinity over time would increase the sharpness and magnitude of the Bragg peak. It should be considered that the *q* resolution of these diffraction data recorded *in situ* on the spin echo spectrometer is limited, not allowing a separation of different structural features (Maier *et al.*, 2021 ▸). Therefore, the results obtained from the diffraction data might average over several contributions.

Interestingly, the microscopy results (Fig. 1 ▸) seem to indicate a smaller *t*_0_ (Table 1 ▸) closer to the neutron spectroscopy values. The reason for this difference in the absolute value of *t*_0_ could arise from the formation of small clusters, which appear static in the diffusive picture prior to becoming visible by microscope.

For *c*_s_ = 4.875 m*M*, the techniques investigating the structural features (microscopy and diffraction) reveal characteristic times Δ*t* ≈ 7 h, which are around 60% smaller than for the techniques probing diffusion (DLS, NSE, NBS) with Δ*t* ≈ 11 h (Table 1 ▸). For the second sample with *c*_s_ = 4.5 m*M*, the differences between NSE and DLS indicate different dynamics happening on the different timescales involved, pointing towards a different pathway compared with the *c*_s_ = 4.875 m*M* salt sample.

Unavoidably, for efficient use of the beamtime, different sample containers have to be used for each acquisition, and specific container types for each technique (see *Materials and methods*[Sec sec2]). Considering that this might cause subtle geometry and surface effects, the results for both *t*_0_ and Δ*t* reported in Table 1 ▸ agree remarkably well between NBS and NSE (data available only for the sample with *c*_s_ = 4.875 m*M*). These results also agree well with the structure inferred *in situ* from NSE [row ‘NSE (diffraction)’ in Table 1 ▸]. In contrast, at low *q* below the structural dynamic range captured by NBS, the structural rearrangements seen in NSE seem to become slower [row ‘NSE (diff. low *q*)’ in Table 1 ▸]. For the *c*_s_ = 4.875 m*M* sample, the timescales approach those seen in DLS when allowing for two sigmoid functions because of the longer DLS experiment duration as explained above. We emphasize that the existing data set is still too small. Therefore, the above discussion at present results in the mere speculation that the results for *t*_0_ do seem to differ depending on the technique, with the growth rate seen in DLS and neutron diffraction lagging behind the rate seen via the nanosecond diffusion probed with neutron spectroscopy.

### 3.8. Comparison with a protein–salt system passing through a metastable liquid phase

Salt-induced protein crystallization has been observed and investigated for several systems. Non-classical crystallization processes forming metastable intermediate phases (MIP) have, for example, been observed for BLG in the presence of CdCl_2_ in D_2_O with SANS (Maier *et al.*, 2021 ▸).

**Figure 8:**
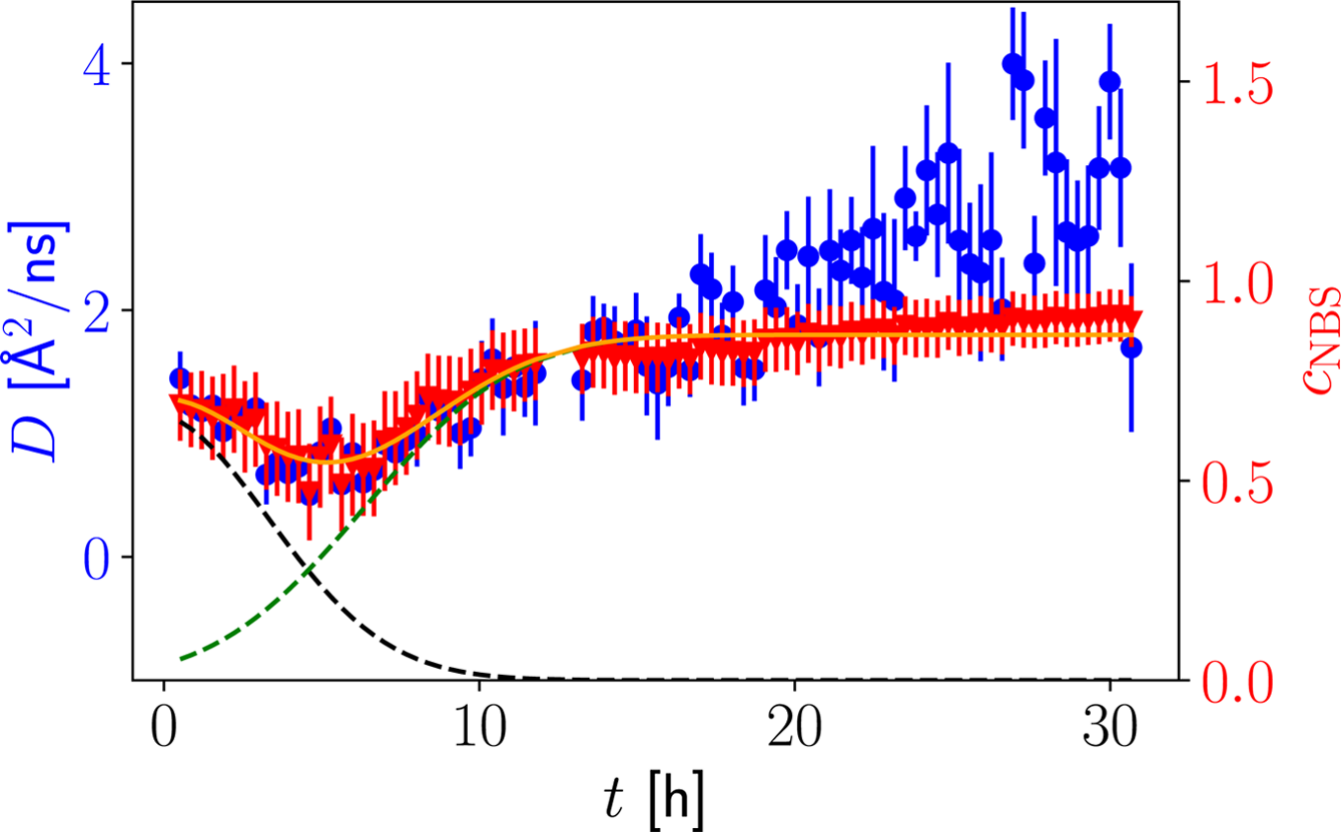
Time-dependent neutron spectroscopy data for BLG (84.4 mg ml^−1^) and CdCl_2_ (30 m*M*) in D_2_O. The blue circles (left axis) describe the short-time self-diffusive properties of BLG as a function of time determined from FWSs. The red triangles (right axis) describe the elastic contribution in the sample which is characterized by a decaying contribution of a dissolving gel-like phase (black dashed line) and an increasing crystal fraction (green dashed line).

We have explored this process by both time-resolved NBS and NSE for BLG, *c*_p_ = 84.4 mg ml^−1^, with *c*_s_ = 30 m*M* CdCl_2_ in D_2_O at ambient temperature. The NBS–FWS data (Beck *et al.*, 2019*a* ▸) were analyzed with the same framework as explained in the previous sections and result in a non-monotonic time dependence of the immobile fraction of the proteins (Fig. 8 ▸). This time dependence can be described by a vanishing contribution of the intermediate phase during the first ∼5 h (black dashed curve) and by a noticeably growing contribution of crystals (green dashed curve) in the sample beyond the first ∼10 h (Fig. 8 ▸). The crystals and the gel-like phase appear as an elastic contribution, and their sum (orange line) can be modeled with the same models used for the kinetic time dependence of the system determined by SANS (Maier *et al.*, 2021 ▸). The observed short-time self-diffusion coefficient initially decays with the vanishing fraction of the MIP, presumably due to the increase in crowding or small clusters. The subsequent increase in the diffusion coefficient is attributed to a dilution of the liquid phase due to the crystal growth, which has been observed previously for ZnCl_2_-induced crystallization of BLG (Beck *et al.*, 2019 ▸).

Time-resolved measurements with neutron spin echo spectroscopy on IN11 (Dagleish *et al.*, 1980 ▸) show no changes in the time evolution of the intermediate scattering function (Fig. S9), although at the end of the measurements, crystals were clearly visible in the quartz capillary (Fig. S10). According to the results from HSA in the presence of LaCl_3_, several aspects might explain the absent changes in the NSE signal. Given the relatively fast kinetics in the BLG–CdCl_2_ system reported in Fig. 8 ▸, the acquisition time of approximately four hours per intermediate scattering function on IN11 might not be fast enough to capture the kinetic changes. Moreover, the investigated (*q*, τ) window might not be suitable for the system investigated, with *q* being too small to approximate self-diffusion and too high to cover the Bragg peaks in the sample. In addition, the smaller collective diffusion might not be captured by the limited echo time.

## 4. Conclusions

We have presented kinetic studies, using several methods, of different salt-induced protein crystallization pathways. As model systems, two combinations of protein and salts (HSA–LaCl_3_ and BLG–CdCl_2_) have been used. For HSA in the presence of LaCl_3_, we showed salt-concentration-dependent crystal structures with SANS measurements and followed the crystallization with kinetic measurements using neutrons (NSE, NBS), dynamic light spectroscopy and microscopy. The highly reproducible and controlled crystallization process of the model protein systems in combination with the good time resolutions of all techniques (defined by the time of the individual iterations; NSE: 2 h; NBS, DLS, microscopy: 30 min) allowed us to obtain comparable datasets with both the neutron spectrometers and complementary experiments and allowed us to disentangle different kinetic processes. For the HSA samples in the presence of LaCl_3_, the kinetic evolution has been described by a sigmoid or logistic function. For each individual condition, the different techniques result in comparable kinetic trends. The comparison of the kinetic evolution of the dynamic data from NSE and structural data from diffraction recorded *in situ* on the same sample reveals slightly different time dependencies. The Bragg peaks seem to evolve more slowly than the elastic contribution, possibly as a result of an internal organization of initially disordered protein aggregates into crystals.

Moreover, we have established a method to quantitatively observe the evolution of the coexistence of protein monomers and small clusters with crystals. For HSA in the presence of LaCl_3_, we find that the average self-diffusion in the dissolved phase decreases slowly with time, indicating a shift of the particle size distribution towards larger clusters. For BLG in the presence of CdCl_2_, the diffusion coefficient initially decreases while the gel-like phase dissolves, resulting in an increase of the local protein concentration. Later, the crystal growth reduces the effective protein concentration and, therefore, accelerates the observable diffusion of the remaining free proteins. By using neutron spectroscopy we are able to distinguish these opposing effects of decreasing crowding (where an increasingly dilute dissolved phase results in increased diffusion) and slow aggregation within the dilute phase (leading to increased elastic scattering) characterizing the crystallization process.

We find fitted kinetic times from all techniques, connected with the associated physically different observables. Even though these characteristic times differ slightly, they nevertheless represent a consistent crystallization behavior across all methods, supporting the picture of a congruent crystallization process on all observation scales. The available data are at present still limited by the availability of neutron beamtime, such that, obviously, more systems will need to be investigated in the future to explore the systematics of crystallization processes.

By uncovering the non-monotonic time dependence of the immobile fraction for the BLG system (Section 3.8), differing from the monotonic time dependence found for the HSA system in the preceding sections, we have illustrated that kinetic measurements involving nanosecond dynamic information can help to discern distinct pathways, as shown from the comparison of Figs. 7 ▸ and 8 ▸. It can be thus inferred whether or not the crystallization passes through a metastable intermediate phase which shows remarkably distinct kinetics. More generally, we can assume that the combination of kinetic measurements observing different length (ångström to micrometre) and timescales (nanoseconds to seconds), jointly with insights on structural properties, will further help a better understanding of future model systems. For both model systems investigated in the present study, we find that the crystallization process is far from instantaneous. Rather, it is limited by diffusive transport and structural rearrangements, as shown by the several-hour sigmoid time dependencies of both the mol­ecular-scale structural and protein center-of-mass diffusion signatures. The quantitatively determined parameters Δ*t* and *t*_0_ as per the model equation (2) are summarized for all methods in Table 1 ▸. Our study has benefited from the advent of the wide-angle spin echo technique using the novel WASP spectrometer, recording structural and dynamic information simultaneously. We have also been able to use the novel approach to obtain absolute values of diffusion coefficients from the ratio of neutron backscattering signals recorded on the IN16B spectrometer by elastic and inelastic fixed window scans. Our method framework could in the future be applied to numerous model systems.

## Supplementary Material

Supporting Information. DOI: 10.1107/S160057672500353X/jo5120sup1.pdf

Video of microscopy images of growing crystals HSA 75mg/ml LaCl3 4.5mM. DOI: 10.1107/S160057672500353X/jo5120sup2.avi

Video of microscopy images of growing crystals HSA 75mg/ml LaCl3 4.5mM, highlighted. DOI: 10.1107/S160057672500353X/jo5120sup3.avi

Video of microscopy images of growing crystals HSA 75mg/ml LaCl3 4.875mM. DOI: 10.1107/S160057672500353X/jo5120sup4.avi

Video of microscopy images of growing crystals HSA 75mg/ml LaCl3 4.875mM, highlighted. DOI: 10.1107/S160057672500353X/jo5120sup5.avi

Data from beamtime 8-04-853: https://doi.ill.fr/10.5291/ILL-DATA.8-04-853

Data from beamtime 8-04-940: https://doi.ill.fr/10.5291/ILL-DATA.8-04-940

Data from beamtime 8-04-862: https://doi.ill.fr/10.5291/ILL-DATA.8-04-862

Data from beamtime 8-04-953: https://doi.ill.fr/10.5291/ILL-DATA.8-04-953

## Figures and Tables

**Table 1:** Kinetic timescales for the two different crystallization conditions (LaCl_3_ salt concentration *c*_s_ = 4.5 and 4.875 m*M*) determined from different time-resolved measurements obtained by fitting equation (2) All samples were at an HSA concentration of *c*_p_ = 75 mg ml^−1^. The parameters for the DLS measurements, given in the top two rows, result from a fit using a sum from two sigmoid functions [equation (2)]. All other entries in the table result from a single sigmoid description. The values in the ‘Microscopy corr.’ row are the parameters from the microscopy results corrected for the crystallite surface-to-volume ratio (see main text and supporting information). The absence of Bragg peaks in the *q* range investigated prevents the analysis with the proposed models for *c*_s_ = 4.5 m*M* (marked with –).

	*t*_0_ (h)		Δ*t* (h)	
	*c*_s_ = 4.5	*c*_s_ = 4.875	*c*_s_ = 4.5	*c*_s_ = 4.875
DLS first	96.93 ± 0.51	60.56 ± 0.98	3.21 ± 0.64	5.94 ± 1.13
DLS second	108.19 ± 1.59	81.46 ± 22.35	14.65 ± 1.04	11.43 ± 7.56

Microscopy	31.92 ± 1.99	28.47 ± 2.14	7.08 ± 1.90	7.32 ± 1.88
Microscopy corr.	90.77 ± 1.94	87.40 ± 2.08	5.99 ± 1.69	6.19 ± 1.67
DLS one sigmoid	102.93 ± 0.45	64.58 ± 0.23	9.06 ± 0.38	7.16 ± 0.20
NBS	Not measured	40.00 ± 0.35	Not measured	11.48 ± 0.16
NSE	–	45.24 ± 1.77	–	11.65 ± 0.84
NSE (diffraction)	–	48.98 ± 0.63	–	7.15 ± 0.45
NSE (diff. low *q*)	25.88 ± 0.58	74.20 ± 2.75	6.17 ± 0.58	13.47 ± 0.70

## Data Availability

Neutron data are permanently curated by the Institut Max von Laue–Paul Langevin and available in the following references: Beck *et al.* (2023) ▸, Mateo Miñarro *et al.* (2023 ▸), Beck *et al. *(2019*a* ▸), Beck *et al.* (2020 ▸). Microscopy and DLS data are available via Beck *et al.* (2023 ▸). The MATLAB code for the analysis of the time-dependent microscope data is available from https://github.com/chribe/Microscope_Crystals.
