# Supplementary material for: A multiscale in situ time-resolved study of the nano- to millisecond structural dynamics during protein crystallization
Source: J Appl Crystallogr. 2025 May 29;58(Pt 3):845–58. doi: 10.1107/S160057672500353X (PMC12135975; doi:10.1107/S160057672500353X)
Supplement: Supplementary file 1 [file j-58-00845-sup1.pdf]

Supporting Information:
A multiscale *in situ* time-resolved study of the nano- to millisecond structural dynamics during protein crystallization

Christian Beck[1,2], Ilaria Mosca[1,2], Laura M. Miñarro[1,2,3], Benedikt Sohmen[1,2], Cara Buchholz[1], Ralph Maier[1], Lara Franziska Reichart[1], Anna Carlotta Grundel[2,4], Famke Bäuerle[1,2], Roody Nasro[1,2], Hadra Banks[1], Simon Christmann[1], Kai-Florian Pastryk[1], Bela Farago[2], Orsolya Czakkel[2], Sylvain Prévost[2], Alexander Gerlach[1], Marco Grimaldo[1,2], Felix Roosen-Runge[5], Olga Matsarskaia[2], Frank Schreiber[1], Tilo Seydel[2]

[1] Institute of Applied Physics, University of Tübingen, 72076 Tübingen, Germany
[2] Institut Max von Laue – Paul Langevin, 38042 Grenoble Cedex 9, France
[3] Université Grenoble-Alpes, 38400 Saint-Martin-d'Hères, France
[4] Fakultät für Physik und Astronomie, Im Neuenheimer Feld 226, 69120 Heidelberg, Germany
[5] Division of Physical Chemistry, Lund University, Naturvetarvägen 22, 22362 Lund, Sweden

1 Samples investigated

Table S1 shows the different sample conditions investigated as well as the corresponding techniques applied.

Sample condition		Technique				
HSA [mg/ml]	$LaCl_3$ [mM]	SANS D33	DLS	Microscopy	NSE WASP	NBS IN16b
50	3	x				
	3.5	x				
	3.75	x				
75	4.875	x	x	x	x	x
	4.5	x	x	x	x	
	3.75	x				
	4.125	x				
	5	x				
	5.25	x				
100	5	x				
	5.5	x				
	6	x				
	6.5	x				
	7	x				

Table S1: Different sample conditions measured on HSA – $LaCl_3$ with different techniques. D33, WASP, and IN16b are the names of the neutron instruments used. All samples have been measured in D_2O.

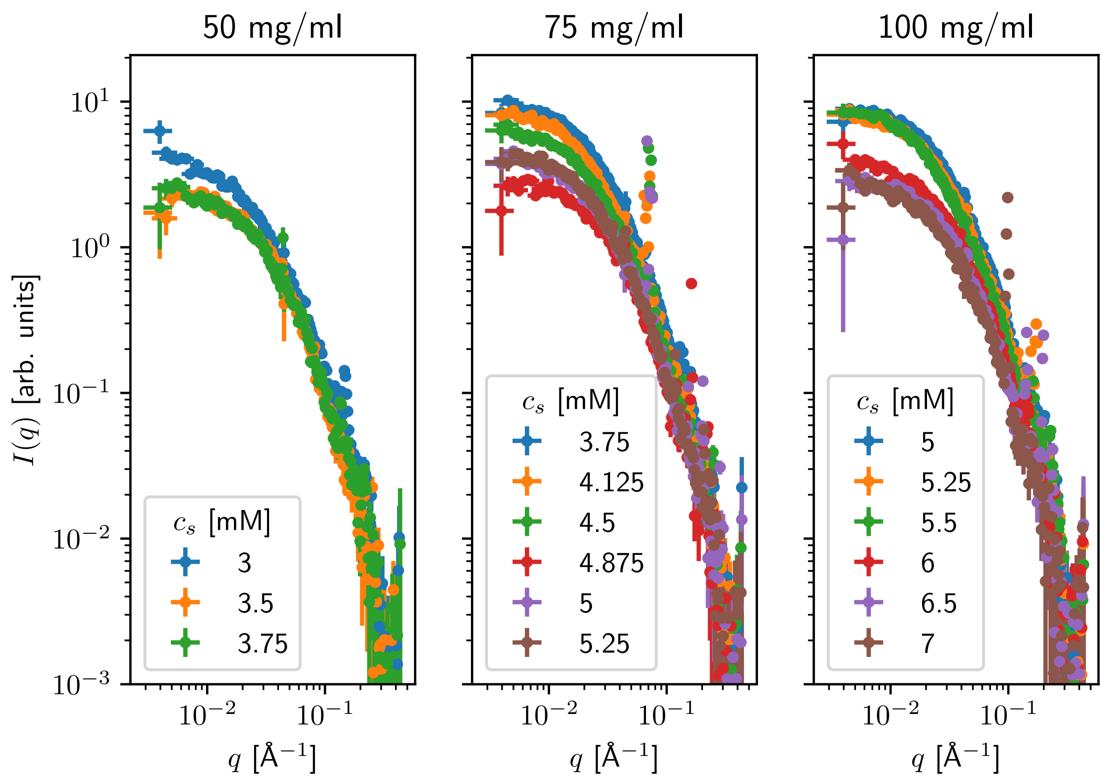

Figure S1: Background-subtracted scattering signal of the SANS measurements acquired on D33 of the investigated samples. Significant changes in the Bragg-peak positions can be observed. The three subplots represent the different protein concentrations (c_p=50, 75, 100 mg/ml) of HSA respectively. The $LaCl_3$ concentration is given in the legend of each plot.

2 Characterization by SANS

The time dependence of the structural evolution of crystal growth as well as the Bragg peak position, and therefore the crystal structure, have been determined on the small-angle neutron scattering beamline D33 (experiment 8-04-953, Ref.[1]). To explore the influence of salt and protein concentration, we compare the SANS profile of the final crystallized sample with the initial state measured directly after preparation. Employing a radial average, the Bragg peak position as well as the overall contribution to the scattering signal of the Bragg peaks can be determined. We describe the coherent part of the signal as a combination of the protein signal of HSA in solution, determined with the first measurement directly after sample preparation, and a contribution from the Bragg peaks caused by the crystals. The scattering function can therefore be written as

$$I_{\text{final}}(q) = a_0 \cdot I_{t=0}(q) + I_{\text{Bragg}}(q) + I_{inc}(q) \tag{1}$$

with the scaling parameter a and the incoherent contribution $I_{inc}(q)$. We use the time independent plateau at high q to fix the incoherent contribution and determine with a fit the scaling parameter a at the low q range of the outer detector panels ($0.05\,\text{Å}^{-1} < q < 0.06\,\text{Å}^{-1}$). Figure S1 displays the background-subtracted scattering signal different protein and salt concentrations. Different Bragg peak positions can be observed depending on the salt concentration.

In Figure S2, the q value of the most pronounced Bragg peak from Figure S1 is displayed as a function of the salt concentration for the different protein concentrations. While for all three protein concentrations investigated, general trends can be observed, significant outliers can be observed within the salt series.

To further investigate the origin of these different crystal structures, we focus here on two conditions at constant protein concentration $c_p = 75$ mg/ml with two salt concentrations ($c_s = 4.5,\ 4.875$ mM) which are

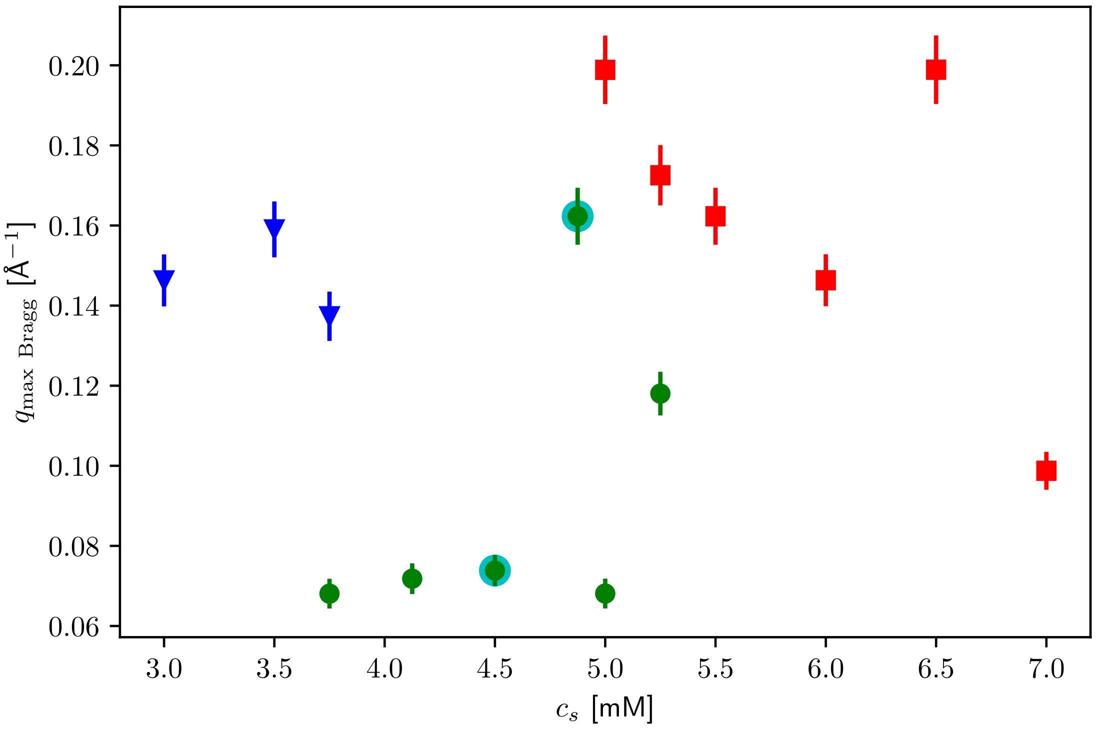

Figure S2: q value of the most pronounced Bragg peak from $LaCl_3$ induced HSA crystals determined from the Bragg peaks visible in Figure S1 as a function of c_s for different protein concentrations. Blue triangles, green circles and red squares represent the samples with 50 mg/ml, 75 mg/ml and 100 mg/ml, respectively. The two sample conditions which are further investigated in this paper are highlighted with a cyan background.

close together but result in different structures. These two conditions are highlighted with cyan background in Figure S2.

3 Additional NSE measurements

The second sample investigated (HSA: $c_p = 75\ \frac{\mathrm{mg}}{\mathrm{ml}}$; $LaCl_3$: $c_s = 4.5$ mM) is shown in Figure S3. In contrast to the previous sample, no significant changes are visible in the intermediate scattering function over time. The diffraction data in the lower subplot shows kinetic changes at low q as further analyzed in the main article.

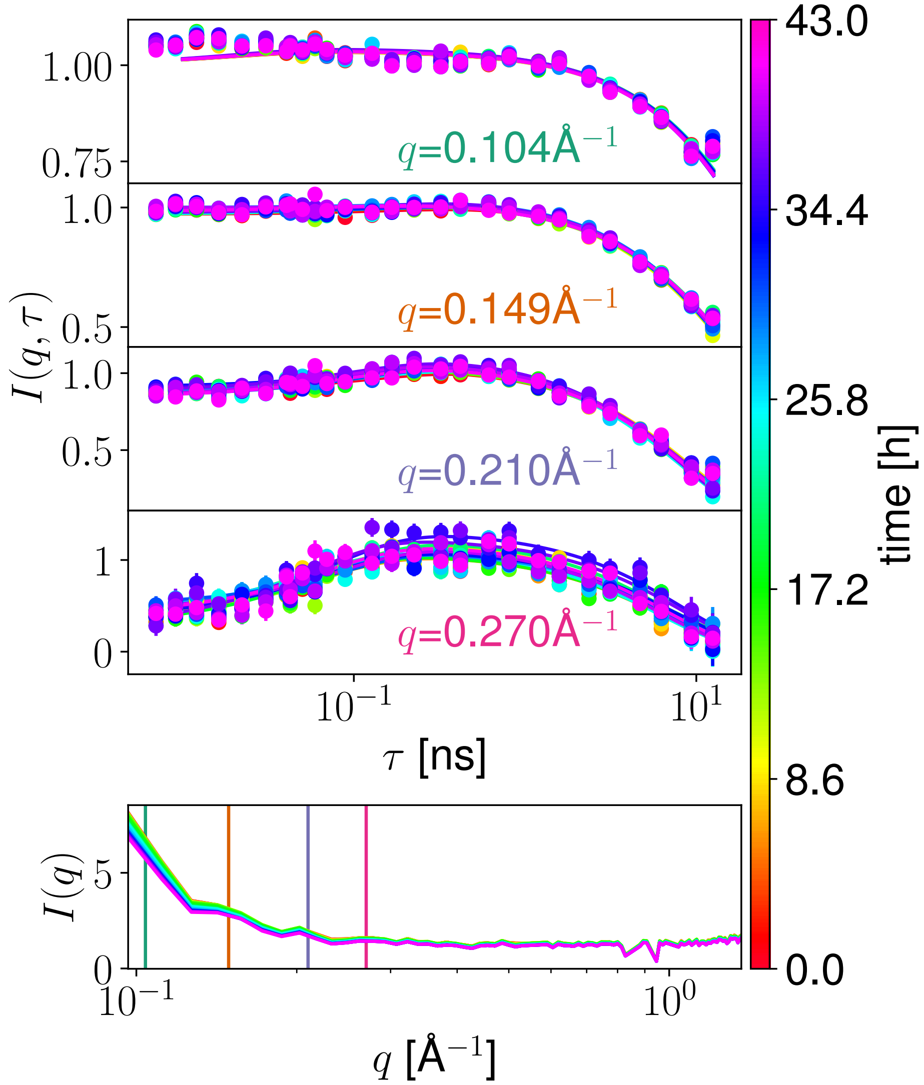

Figure S3: kinetic time-dependent WASP data of HSA 75 mg/ml and $LaCl_3$ c_s=4.5 mM. While the kinetic data do not show changes during the crystalization process, the decaying diffraction signal in the lowest plot indicate the crystal growth progress.

4 Optical Microscopy

Similar to the sample shown in the main article, the HSA sample with 4.875 mM $LaCl_3$ has also been investigated by time-resolved optical microscopy (see Figure S4). Similar analysis frameworks as for the first samples have been applied and comparable results have been found. However, since the overall number of crystals observed is smaller, the resulting histograms quantifying the parameters are not as precise as in the first case.

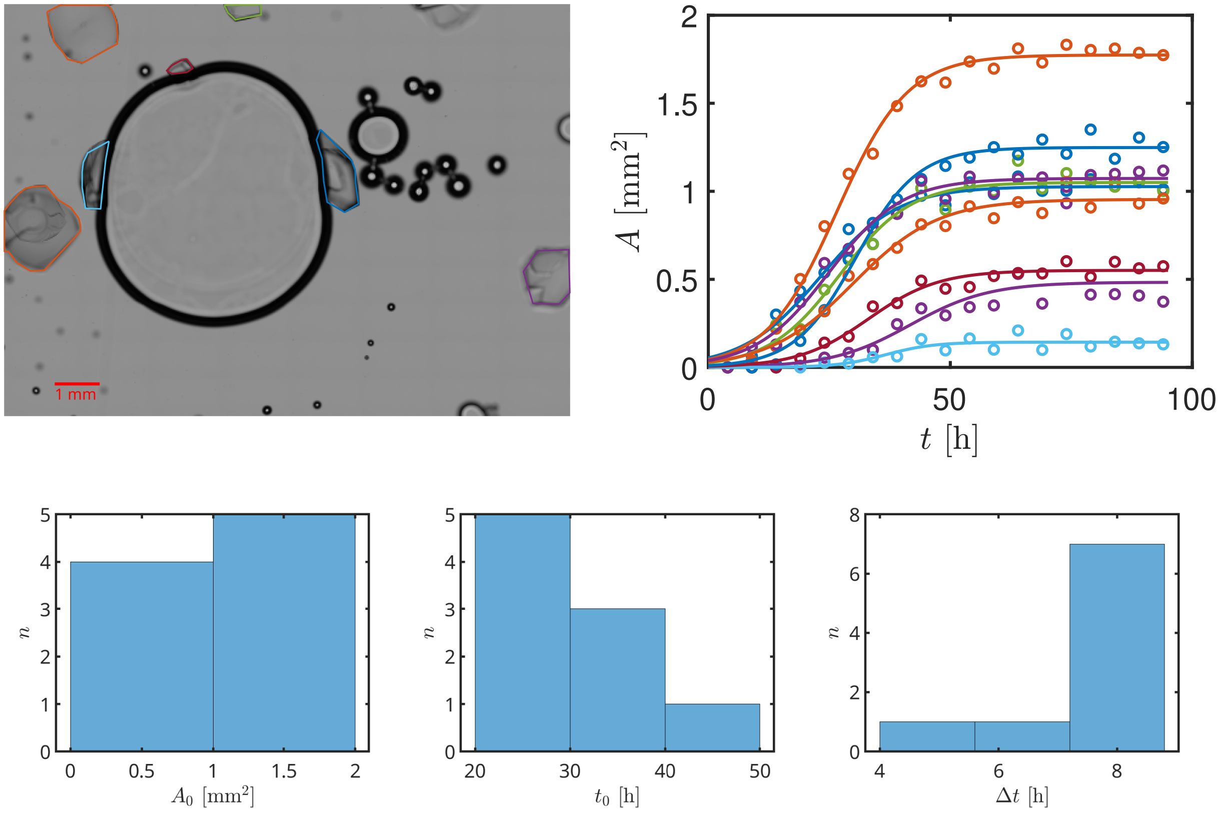

Figure S4: Microscopy analysis for the sample HSA $c_p = 75$ mg/ml with $c_{LaCl_3} = 4.875$ mM.

5 Crystallite face area to volume conversion in optical microscopy

Assuming the volume of the crystal versus time t can be described by a sigmoid function $v(t)$, the observed surface (observable 2D projected area) should scale as $s(t) = (v(t))^{2/3}$. Figure S5 below shows the time dependence of the sigmoid $s(t)$, the edge length as well as the volume, assuming $t_0 = 0$, $\Delta t = 1$. As can be seen, all three curves show similar increases. For a systematic investigation, we describe the time dependence of $s(t)$ by a sigmoid function for different values of Δt. In Figure S6, the fit result Δt_s of $s(t)$ is depicted versus the assumed Δt_v for $v(t)$. It is visible that Δt_s increases faster than Δt_v and can be described by $\Delta t_s = 1.205 \cdot \Delta t_v - 0.1340$. If this equation is assumed to correct for the volume versus surface effect, the microscopy values of Δt are in even better agreement with the diffraction values of NSE for $c_s = 4.5$mM, while for the second concentration, the values obtained by microscopy are a factor two smaller than the ones determined by NSE. For t_0, a correction can be obtained by $t_{0s} = 1.025 t_{0v} - 61.12$.

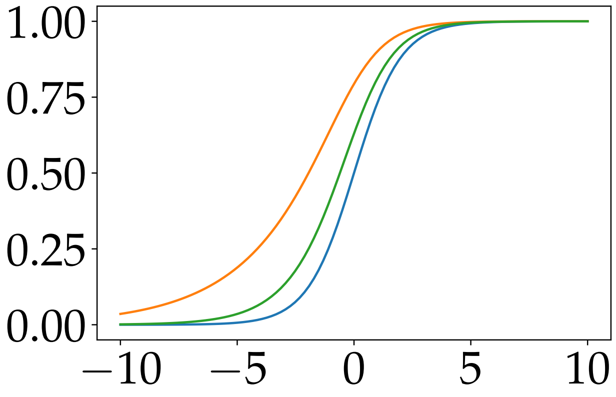

Figure S5: Time dependence of the crystal volume assumed as a sigmoid function (blue line). The resulting length of the crystallite edge l (orange line) has been defined by $l(t) = v(t)^{1/3}$. The square of this edge length $l^2(t)$ is taken to describe the time-dependence of the surface (green line).

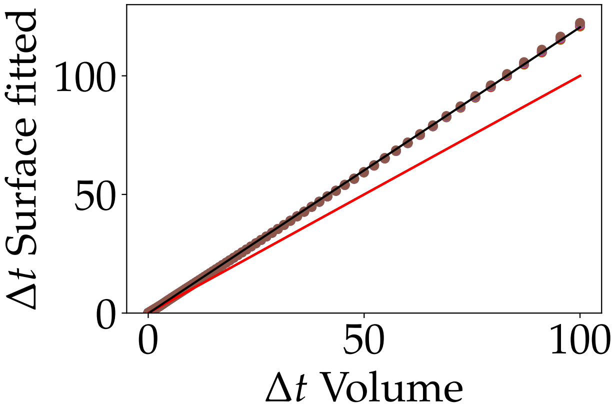

Figure S6: The values Δt_s from the fits of the crystallite face area versus the assumed Δt_v for the crystallite volume. It becomes apparent that the obtained Δt_s is larger than the bisector (red line) and, therefore, larger than the assumed Δt_v. The dependency can be described by $\Delta t_s = 1.205 \cdot \Delta t_v - 0.1340$. The analysis has been carried out for different values of t_0. These fits are represented by symbols with different colors in the plot that coincide perfectly, such that they cannot be discerned.

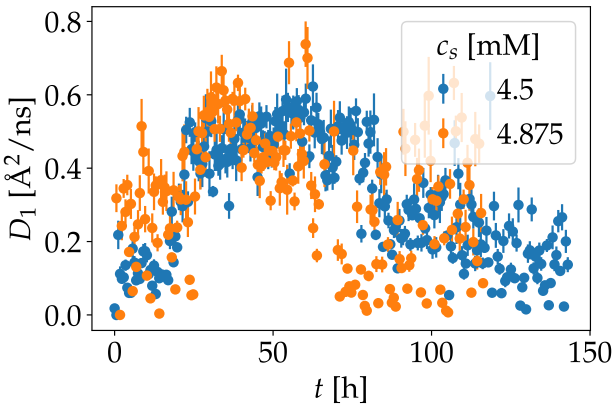

Figure S7: Diffusion coefficients of the clusters determined from the time resolved DLS measurements of HSA (75 mg/ml) in the presence of $LaCl_3$ with c_s =4.5 mM (blue) and c_s =4.875 mM (orange). Both samples are characterized by an initial increase of the diffusion coefficients over time. The later decrease of the diffusion coefficients can be associated with the formation of bigger clusters and aggregates.

6 DLS measurements

The diffusion coefficients of the clusters or small crystallites as a function of time are shown for both HSA samples investigated in Figure S7.

7 Kinetic time dependence of DLS data on restricted time range

To test the assumption of one single sigmoid function on a restricted range in the kinetic time, we have applied a fit to the data between 40h and 80h (dashed-dotted lines in Figure S8b). With this approach, we obtain:

(a) t_0 [h]

	$c_s = 4.5$ mM	$c_s = 4.875$ mM
DLS (single restrained sigmoid)	73.70 ± 1.79	61.51 ± 0.25

(b) Δt [h]

	$c_s = 4.5$ mM	$c_s = 4.875$ mM
DLS (single restrained sigmoid)	3.46 ± 0.94	6.46 ± 0.29

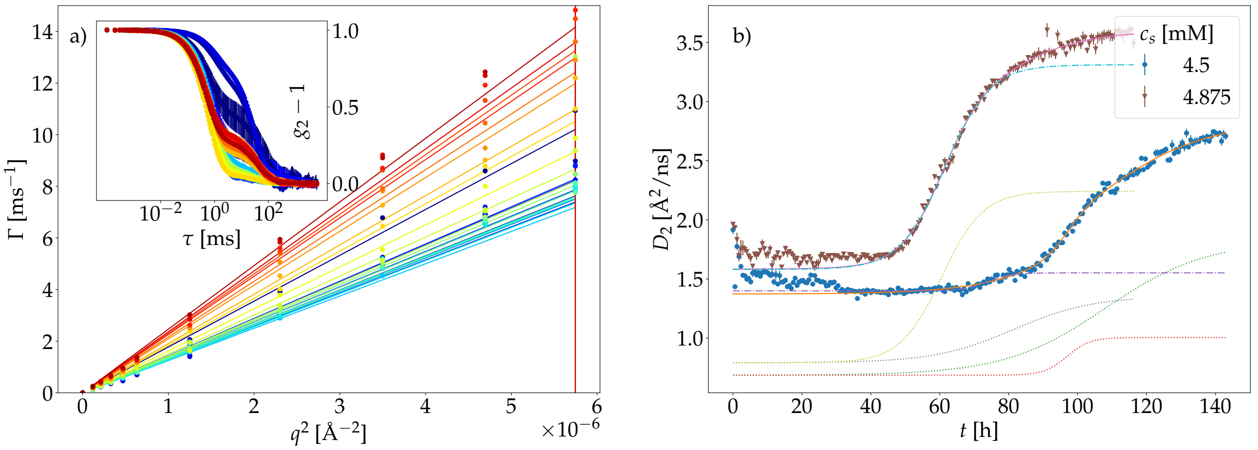

Figure S8: Time-dependent results from the dynamic light scattering (DLS) measurements on HSA $c_p = 75\,\mathrm{mg/ml}$ with $c_s = 4.5$ mM $LaCl_3$ in D_2O. The inset of the left plot (a) displays example DLS correlation functions acquired at different times during the crystallization, fitted by Equation 2 allowing for a sum of two decays. The main part of (a) shows the q-dependence of the decay rates (symbols) for the faster decay Γ_2 of these two fitted contributions. The time dependence is color coded (blue to red) showing each 10th data set. The decay rates are fitted by $\Gamma_i = D_i^{\mathrm{DLS}} q^2$ (solid lines). The right plot (b) displays the kinetic evolution of the fast diffusion coefficients D_2 from DLS, attributed to the protein monomers, for the two samples investigated (symbols), described by a sum of two sigmoid functions, Equation 1 (solid lines). The individual sigmoid contributions are represented by dotted lines. Dashed-dotted lines represent fits to the data between 40h and 80h with one single sigmoid function.

8 Kinetic NSE measurements of $CdCl_2$-induced BLG crystallization

Kinetic NSE measurements have been performed on BLG solutions containing $CdCl_2$ on IN11 during beamtime 8-04-862.[2] The time dependent intermediate scattering functions of the sample (BLG 84.4 mg/ml, $CdCl_2$: 30 mM in D_2O) are shown in Figure S9. Similar to the sample of HSA 75 mg/ml $LaCl_3$ 4.5 mM, the intermediate scattering function is not characterized by kinetic changes. The presence of an initial gel-like phase (see main article) and the consequently growing crystals results in an elastic scattering signal of the sample during the whole acquisition time. Microscopic investigations after the process show crystals in the samples (see Figure S10).

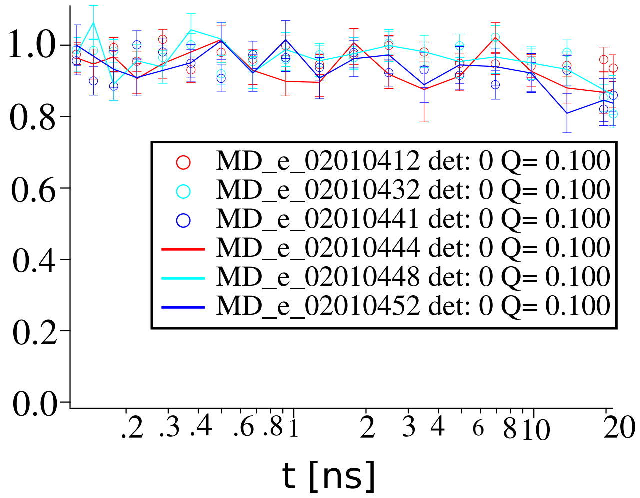

Figure S9: Time-dependent intermediate scattering functions recorded on IN11 during experiment 8-04-862 [2] for BLG 84.4 mg/ml, $CdCl_2$: 30 mM in D_2O at $q = 0.1$ Å^{-1}. No kinetic changes are visible in the intermediate scattering function during the crystallization process and the sample appears static at the investigated time and length scale.

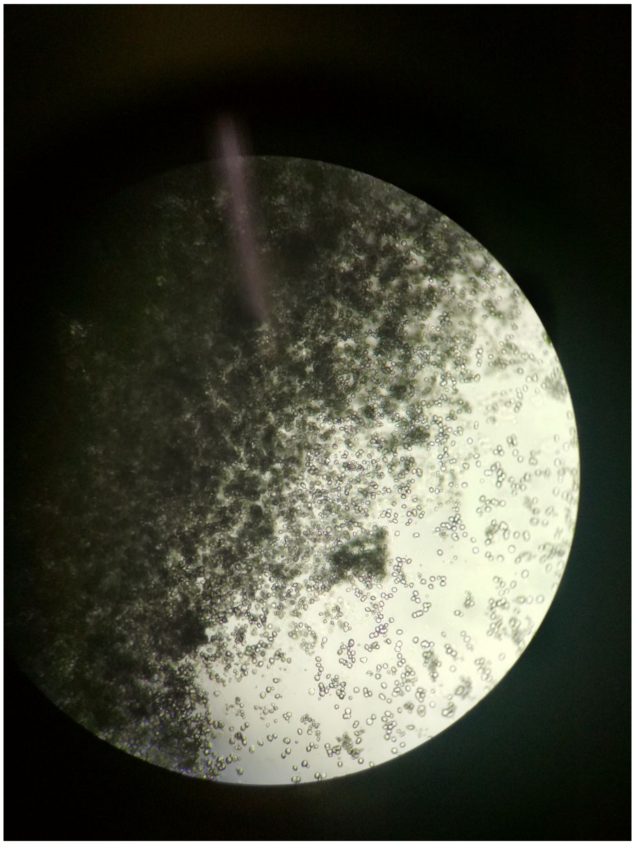

Figure S10: Microscope image of the BLG Sample (BLG 84.4 mg/ml, $CdCl_2$: 30 mM in D_2O) after the NSE measurements with IN11 shown in figure S9. Many protein crystals can be identified in the sample.

References

[1] Mateo Miñarro L, Beck C, Chakraborty S, Devos J M, Grundel A, Laux V, Matsarskaia O, Morozova T, Mosca I, Moulin M, Nasro R, Roosen-Runge F, Schreiber F and Seydel T 2023 Structural and diffusive properties of intrinsically disordered proteins in a polydisperse environment institut Laue-Langevin (ILL): doi:10.5291/ILL-DATA.8-04-953 URL https://doi.ill.fr/10.5291/ILL-DATA.8-04-953

[2] Beck C, Baeuerle F, Czakkel O, Girelli A, Grimaldo M, Maier R, Matsarskaia O, Roosen-Runge F, Schreiber F, Seydel T and Zhang F 2020 In situ real-time study of the diffusive dynamic arrest of proteins during crystallization URL https://doi.ill.fr/10.5291/ILL-DATA.8-04-862
